# Subsuming the Metal Seed to Transform Binary Metal Chalcogenide Nanocrystals into Multinary Compositions

**DOI:** 10.1021/acsnano.1c11144

**Published:** 2022-05-20

**Authors:** Nilotpal Kapuria, Michele Conroy, Vasily A Lebedev, Temilade Esther Adegoke, Yu Zhang, Ibrahim Saana Amiinu, Ursel Bangert, Andreu Cabot, Shalini Singh, Kevin M Ryan

**Affiliations:** † Department of Chemical Sciences and Bernal Institute, University of Limerick, V94T9PX Limerick, Ireland; ‡ Department of Physics and Energy and Bernal Institute, University of Limerick, V94T9PX Limerick, Ireland; § Department of Materials, Royal School of Mines, Imperial College London, Exhibition Road, London SW7 2AZ, United Kingdom; ∥ Catalonia Institute for Energy Research—IREC, 08930 Barcelona, Spain; ⊥ ICREA, 08010 Barcelona, Spain

**Keywords:** nucleation, crystallization mechanism, seed mediated growth, heterostructure, nanorod, metal chalcogenide, thermal conductivity

## Abstract

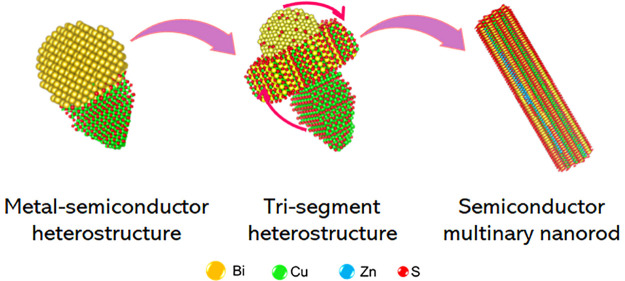

Direct colloidal synthesis of multinary metal chalcogenide nanocrystals typically develops dynamically from the binary metal chalcogenide nanocrystals with the subsequent incorporation of additional metal cations from solution during the growth process. Metal seeding of binary and multinary chalcogenides is also established, although the seed is solely a catalyst for nanocrystal nucleation and the metal from the seed has never been exploited as active alloying nuclei. Here we form colloidal Cu–Bi–Zn–S nanorods (NRs) from Bi-seeded Cu_2–*x*_S heterostructures. The evolution of these homogeneously alloyed NRs is driven by the dissolution of the Bi-rich seed and recrystallization of the Cu-rich stem into a transitional segment, followed by the incorporation of Zn^2+^ to form the quaternary Cu–Bi–Zn–S composition. The present study also reveals that the variation of Zn concentration in the NRs modulates the aspect ratio and affects the nature of the majority charge carriers. The NRs exhibit promising thermoelectric properties with very low thermal conductivity values of 0.45 and 0.65 W/mK at 775 and 605 K, respectively, for Zn-poor and Zn-rich NRs. This study highlights the potential of metal seed alloying as a direct growth route to achieving homogeneously alloyed NRs compositions that are not possible by conventional direct methods or by postsynthetic transformations.

## Introduction

Multinary metal chalcogenide nanocrystals (NCs) with unique chemical and physical properties have enabled significant advances in optoelectronics, magnetics, catalysis, and thermoelectrics.^1−7^ The wet chemical pathways to these NCs are typically initiated with colloidal nucleation of a binary metal chalcogenide seed followed by the inclusion of the additional metal cations in a sequential process.^8−10^ Especially, in the multielemental systems containing Cu^+^ or Ag^+^, the evolution in composition from binary to multinary is often accompanied by a shape change for example, from 0D to 1D enabled by the preferred crystal phase and growth pathways.^9,11,12^ The direct route has not been universally applicable to all possible combinations of metal cations and an alternative approach involves secondary processes where topotactic cation exchange of binary metal chalcogenides NCs postsynthesis is carried out.^13,14^ The cationic diffusion is facilitated by favorable ligand–cation interactions in the host lattice to create vacant sites. For example, aryl and alkyl phosphine ligands have been used for Cu^+^ extraction from a Cu_2-x_S host lattice, enabling Ga^3+^ and In^3+^ incorporation into the vacant sites to form a Cu_2–*x*_S–Cu(Ga and/or In)S_2_ heterostructure prior to the alloyed Cu(Ga and/or In)S_2_ formation.^15^

Shape anisotropy of semiconductor NCs (*e.g.*, nanorods or nanowires) is attractive for the possibility of independent tuning of diameter and length dependent properties and subsequent assembly *via* vertical alignment to form superstructures with enhanced optical and catalytic properties.^16−18^ Additionally, free charge carrier mobility along the long axis results in efficient charge separation and extraction in anisotropic semiconductor NCs.^19,20^ However, anisotropic growth of some multinary NCs (*e.g.*, Cu_3_SnE_4_, CuBiE_2_, Cu_2_ZnSnE_4_, E = S, Se) is not feasible using homogeneous colloidal nucleation or postsynthesis cation exchange processes. For these, seeded approaches have proven attractive where solely ion-conducting metal chalcogenide seeds such as Ag_2_E (E = S, Se), Cu_2–*x*_S have been employed as catalysts in diffusion-controlled growth mechanisms.^21−24^ The high mobility of the seed cations creates vacant sites for foreign cation incorporation. Subsequent supersaturation promotes NC growth where alloying of the growth phase and seed is feasible depending on the charge balance of coordination sites. There is also another less studied route to materialize multicomponent metal chalcogenide 1D nanostructures, where a liquid metal droplet is used to catalyze the desired semiconductor phase in a solution–liquid–solid (SLS) growth mechanism.^20,25^ This approach has been long studied for covalent network nanowires of Si, Ge but has also been used to form metal-seeded CuInE_2_, ZnE, CdE (E = S, Se, Te) nanowires.^26−31^ The seed here is a catalyst to lower the eutectic temperature and at the end of the reaction remains intact as a metal particle either coupled to the semiconductor component as a heterostructure or separated in solution.^32−34^

In this work, we generated multinary Cu–Bi-based chalcogenide crystal phases which display intrinsically low lattice thermal conductivity. Despite the potential of these phases for application in thermoelectric devices, thermal barrier coatings, and rewritable data storage,^35,36^ they are sparsely explored as colloidal NCs because of challenges in finding an optimal window where the kinetic and thermodynamic parameters modulate the reactivity of cationic and anionic precursors to avoid secondary phase formation of Bi_2_E_3_ and Cu_*x*_E.^37,38^ Herein, the *in situ*-formed Bi nanoparticles (NPs) catalyzed the heterogeneous nucleation of Cu_2–*x*_S forming Bi–Cu_2–*x*_S heterostructures contrary to the nucleation of Cu_2–*x*_E as observed in conventional multinary Cu chalcogenide NCs. We demonstrate that the incorporation of Cu and sulfurization transform the metallic bismuth seed into a trisegmented heterostructure with a Bi-rich Bi_*x*_Cu_*y*_S_*z*_ phase, a Cu-rich Bi_*x*_Cu_*y*_S_*z*_ stem, and an alloyed transitional Bi_*x*_Cu_*y*_S_*z*_ segment present at the heterointerface. We find that the compositionally homogeneous nanorods (NRs) formation is facilitated by the transformation of the trisegmented heterostructure *via* gradual dissolution of the Bi-rich seed and recrystallization of the Cu-rich stem into the transitional segment. The growth kinetics of this process explains how the formation of a metal seed that bypasses the binary metal chalcogenide nucleation can be a gateway to form complex compositions with homogeneous elemental distribution. We also investigated how the incorporation of Zn affects the aspect ratio resulting from axial elongation of the NRs. Furthermore, we studied the thermoelectric properties of these multinary NRs. On the basis of the Zn concentration, the NRs can exhibit different transport properties (n- or p-type), and low thermal conductivity values that are promising as intrinsically low thermal conductive materials.

## Results and Discussion

Cu–Bi–Zn–S nanorods were prepared using a colloidal hot-injection approach where Cu(acac)_2_, BiCl_3_, and ZnCl_2_ were used as cationic precursors in the presence of oleylamine (OLA) with octadecene (1-ODE) and trioctylphosphine oxide (TOPO) as solvents. A mixture of *tert*-dodecylmercaptan and 1-dodecanethiol was used as the sulfur source and injected into the solution of cationic precursors and solvent mixture at 135 °C with subsequent heating to 250 °C (detailed procedure described in the Experimental Section). To avoid Cu_2-x_S formation on its own, the injection temperature was kept below 140 °C. To understand the growth kinetics and nucleation, aliquots were withdrawn at different temperatures and times and analyzed.

### Bi-Seeded Cu_2–*x*_S Formation

**Figure 1:**
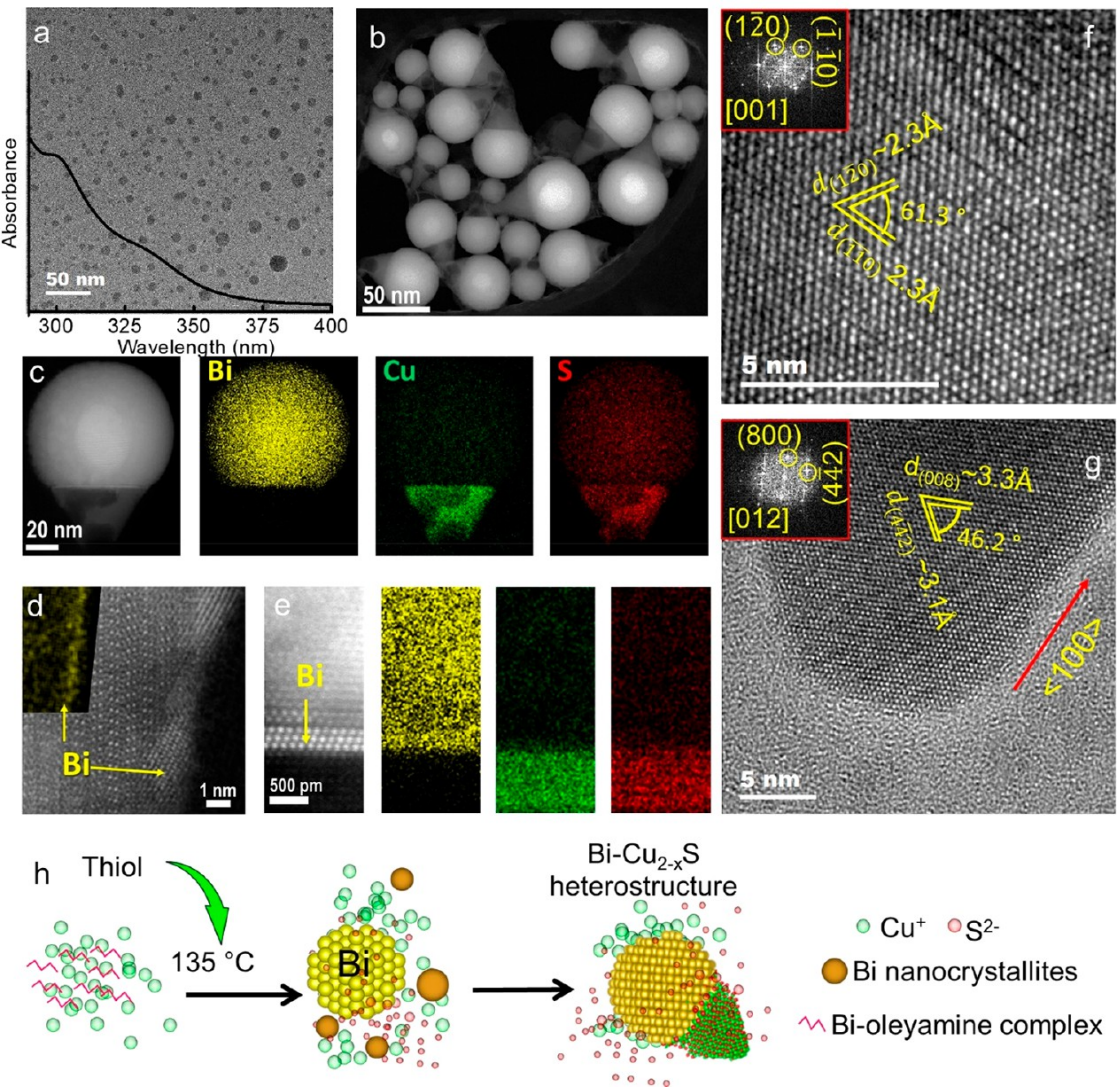
TEM micrograph of Bi NPs overlaid with its UV absorption spectra, (b) Scanning transmission electron microscopy (STEM) annular dark-field (ADF) micrograph of Bi–Cu_2–*x*_S heterostructures derived from a 150 °C aliquot accompanied by (c) STEM-EDS element maps for Bi (yellow), Cu (green), and S (red). (d) Atomic resolution STEM-ADF micrograph of the Cu_2–*x*_S stem from a 170 °C aliquot. The arrow indicates the Bi-rich layer demonstrated in a color representation in the inset. (e) ADF-STEM micrograph of Bi–Cu_2–*x*_S heterostructures from the 150 °C aliquot with STEM energy dispersive X-ray spectroscopy (EDS) element maps for Bi (yellow), Cu (green), and S (red), showing the Bi inclusion in the stem segment. HRTEM images of (f) the Bi seed and (g) the Cu_2–*x*_S stem of Bi–Cu_2–*x*_S heterostructures derived from the 150 °C aliquot accompanied by selected area fast Fourier transform (FFT) pattern in the inset. (h) Schematic depiction of the heteronucleation of Cu_2–*x*_S on *in situ*-formed Bi NPs forming Bi–Cu_2–*x*_S heterostructures.

The reaction starts with the formation of Bi NPs upon addition of thiol and a concomitant color change in the reaction solution to orange from green. The low-resolution (LR) bright-field transmission electron microscopy (TEM)analysis (Figure 1a) of the aliquot withdrawn at 140 °C confirmed the presence of Bi NPs with an average diameter of 6 ± 3 nm (see the size distribution in Figure S2a). The smaller Bi NPs tend to form larger ones in rapid succession because of an in-solution ripening process (Figure S2c,d). The UV absorption spectra (Figure 1a) of the aliquot at 150 °C displayed bimodal excitonic features from Bi NPs, suggesting a disparity in the size distribution of the Bi seeds due to the ripening. The Bi NPs undergo heteronucleation to form Bi–Cu_2–*x*_S heterostructures (Figure 1b) around 150 °C, as evidenced from the color change in reaction solution to black. The scanning transmission electron microscopy–energy dispersive X-ray spectroscopy (STEM EDS) elemental mapping of the nanostructures (Figure 1c) displays the presence of a Cu- and S-rich stem and a Bi-rich head, forming a cone-shaped Bi–Cu_2–*x*_S heterostructure with a Bi-rich thin layer around the sidewalls of the Cu_2–*x*_S stem (Figure 1d). The inclusion of Bi into the stem segment formed a narrow-alloyed region near the heterointerface (Figure 1e). In the heterostructure, the atomic structure of the head predominantly matches with the rhombohedral phase of Bi (Figure 1f). The *d*-spacing calculated from the selected area FFT (Figure 1f, inset) for the (1**2̅**0) and (**1̅1̅0**) facets of the seed segment are 2.3 Å, matching well with metallic Bi. The stem matches with the monoclinic djurleite phase. The *d*-spacing calculated from the high-resolution transmission electron microscopy (HRTEM) and the corresponding FFT (Figure 1g, inset, and Figure S4) for the (4**4̅**2) and (008) facets of the Cu_2–*x*_S segment are 3.1 and 3.3 Å, respectively, with a growth direction along the ⟨100⟩ plane. The X-ray diffraction (XRD) pattern of the heterostructures (Figures S3, S25, and S26) displayed characteristic peaks of the rhombohedral Bi phase (JCPDS No. 04-003-1496). Hence, the heteronucleation of Cu_2–*x*_S on *in situ*-formed Bi NPs generates metal–semiconductor heterostructures of Bi–Cu_2–*x*_S (Figure 1h).

### Bi-Seeded Transformation and Cation Diffusion into the Cu_2–*x*_S Stem

**Figure 2:**
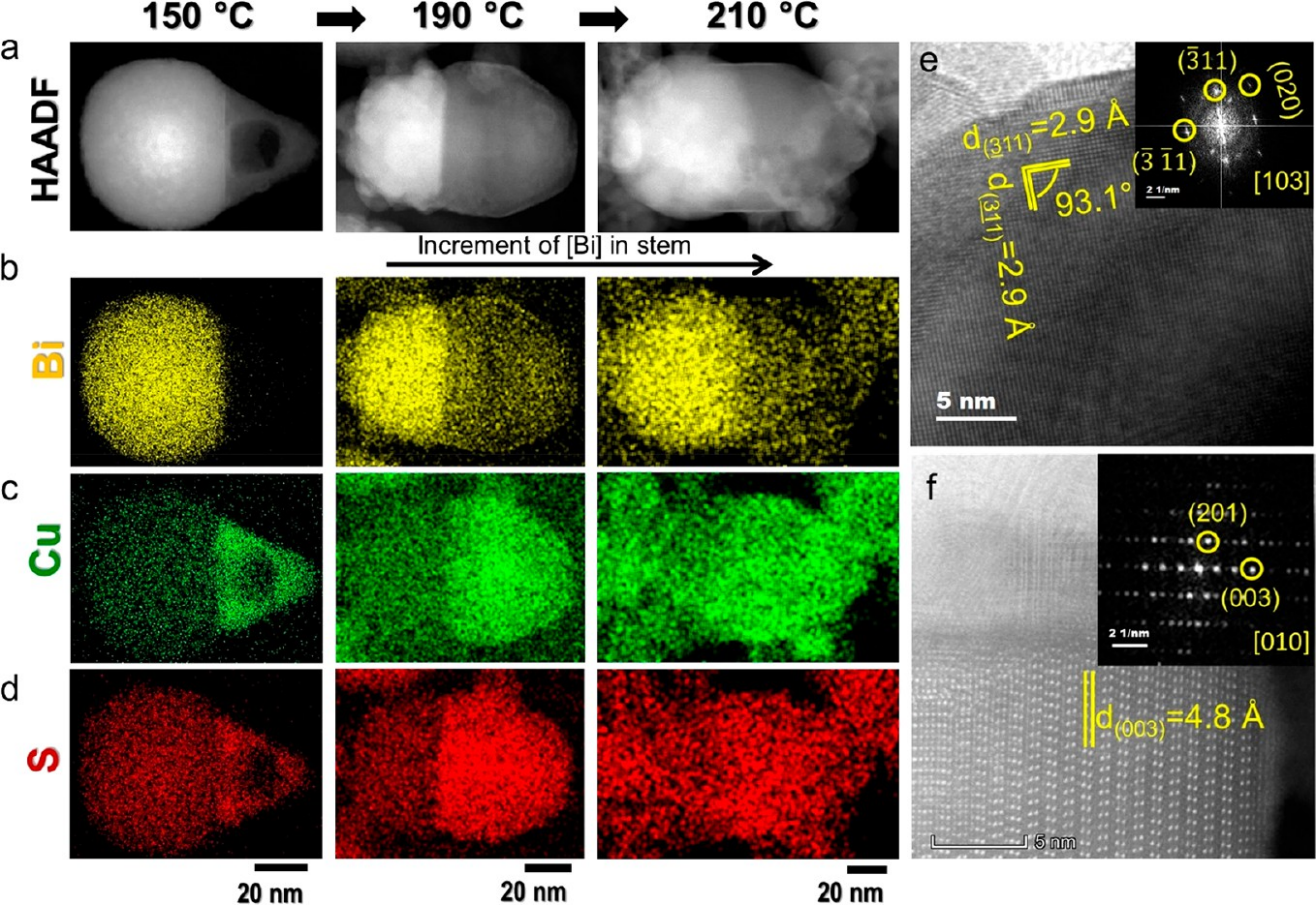
(a) STEM-ADF micrographs of Bi–Cu_2–*x*_S heterostructures derived from the aliquots collected at 150, 190, and 210 °C accompanied by STEM-EDS element maps for (b) Bi, (c) Cu, and (d) S. (e) HRTEM of the seed for a heterostructure derived from a 210 °C aliquot. (f) High-resolution ADF-STEM micrograph of the stem of the heterostructure derived from a 210 °C aliquot accompanied by FFT.

As the temperature is increased to >190 °C, the Bi concentration increases in the stem of the heterostructures (ADF, Figure 2a; STEM-EDS Figure 2b) with the associated transformation of Cu_2–*x*_S into Bi_*x*_Cu_*y*_S_*z*_. Besides, an enlargement of the stem along the short axis can be seen with the increment of Bi concentration. Although the Cu and S signal is most intense in the stem for the NCs, the seed also displays the presence of Cu and S (Figure 2c,d). The incorporation of Cu^+^ and S^2–^ into the seed seemingly changed the rhombohedral metallic Bi to a Bi_*x*_Cu_*y*_S_*z*_ phase. This is confirmed by X-ray photoelectron spectroscopy (XPS) analysis of the NCs where aliquots collected at 150 and 210 °C exhibited peaks near ∼156.5 and ∼161.8 eV originating from Bi^0^ of the seed. However, the peaks near ∼163.8 and ∼158.9 eV from Bi^3+^ of the Bi–S bond of Bi_*x*_Cu_*y*_S_*z*_ phase for the 210 °C aliquot is absent from the 150 °C aliquot suggesting the occurrence of a phase transformation (Figure S31a). The transformation was further confirmed through absorption spectra analysis of the aliquots. The absorption peak (Figure S7) from the Bi NPs at 302 nm diminished at temperatures above 190 °C and an absorption peak emerged around 900 nm from the ternary Bi_*x*_Cu_*y*_S_*z*_ phase. The transformation of the Bi seed into CuBi_5_S_8_ was further demonstrated by HRTEM analysis of an aliquot collected at 210 °C (Figure 2e). The *d*-spacings of 2.1 and 2.9 Å for (020) and (3̅1̅1) planes, respectively, and the angle of ∼92° between (3̅1̅1) and (3̅11) are in conformity with the monoclinic CuBi_5_S_8_ phase. As shown in Figure S5, the selected area FFT from the inner segments of these seeds displays strong satellite intensities from rhombohedral Bi alongside the main peaks from the monoclinic CuBi_5_S_8_ phase, suggesting the transformation of the metallic Bi seeds into a ternary CuBi_5_S_8_ phase. It is also evident from the XRD (Figure S3, S28) of the aliquot at 210 °C that a monoclinic CuBi_5_S_8_ phase formed for which a characteristic peak at 25.5° emerged. Figure 2f displays the STEM-ADF micrograph and the corresponding FFT of the stem section of the heterostructure from the aliquot at 210 °C. In the stem segment, the (003) plane exhibits a *d*-spacing of 4.8 Å while that of the (201) displays a *d*-spacing of 5.7 Å which closely match monoclinic (*C*2/*m*) Cu_2.94_Bi_4.8_S_9_. The growth of the stem occurs along the ⟨100⟩ direction similar to the heterostructures collected below 210 °C. In the XRD of the particles obtained from the 210 °C aliquot the characteristic peaks from the Cu_2.94_Bi_4.8_S_9_ phase emerged at 2θ values of 25.9 and 27.5° corroborating that observation.

### Growth of the Transitional Segment

**Figure 3:**
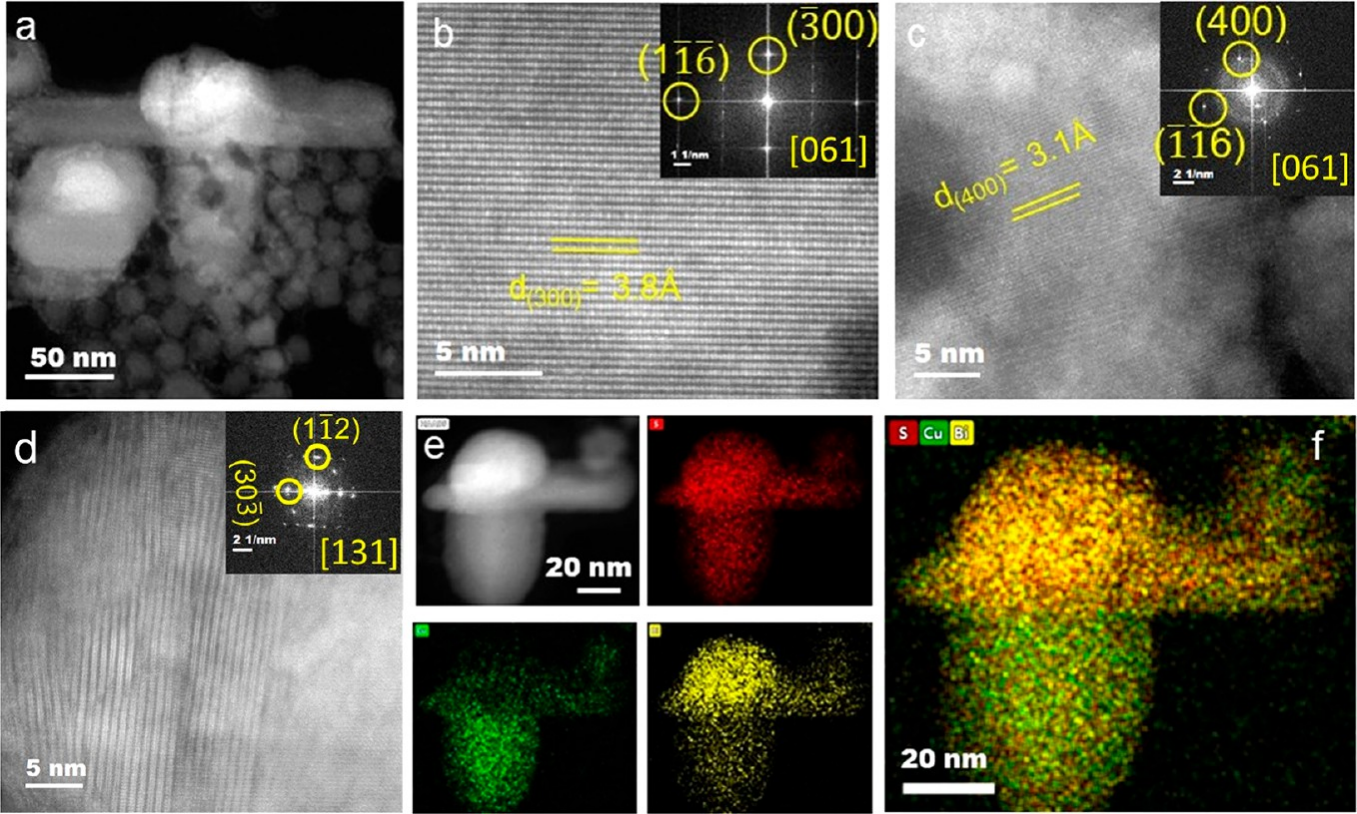
(a) STEM-ADF of the trisegmented heterostructure derived from a 250 °C aliquot and STEM-ADF with selected area FFT of (b) the transitional segment (TS) viewed from the [06̅1] zone axis, (c) the stem viewed from the [061] zone axis, and (d) the seed viewed from the [131] zone axis. (e) STEM-ADF of the trisegmented heterostructure derived from a 230 °C aliquot with the EDS elemental maps for Bi (yellow), Cu (green), and S (red) along with the (f) overlay of the elemental maps.

A transitional segment forms after 230 °C at the heterointerface between the seed and stem creating a trisegmental heterostructure (Figure 3a). The transitional segment is confirmed as Cu_2.94_Bi_4.8_S_9_ monoclinic phase from selected area FFT analysis of the heterointerface of the NCs resulting from the aliquot at 230 °C (Figure S9) and 250 °C (Figure 3b). The transitional segment materialized along the ⟨11̅0⟩ directions with *d*-spacings of 2.1 and 3.8 Å for the (11̅6̅) and (3̅00) planes, respectively, and an angle of ∼91° between the planes, correlating well with that of monoclinic Cu_2.94_Bi_4.8_S_9_ (Figure 3b). Whereas the selected area FFT analysis of the stem segment (Figures 3c and S10) shows *d*-spacings of 4.4, 3.8, 3.5, and 3.2 Å for the (202), (1̅12), (1̅1̅2), and (400) planes, respectively, and an angle of ∼87° between (1̅1̅6) and (400) planes matching with the monoclinic Cu_2.94_Bi_4.8_S_9_, the seed transforms into the monoclinic CuBi_5_S_8_ phase as confirmed from the *d*-spacing of 3.1 Å for the (11̅2) plane (Figure 3d). Panels e and f of Figure 3 exhibit the STEM-EDS elemental mappings for Cu, Bi, and S of the heterostructure with a transitional segment collected at 230 °C. The Cu signal from the stem shows comparatively higher Cu concentration in the stem segment, suggesting the presence of a Cu-rich monoclinic Bi_*x*_Cu_*y*_S_*z*_ phase. The seed with an intense Bi signal at the initial stages of the transitional phase growth transforms significantly into CuBi_5_S_8_ with reduced size as the growth progresses, which is also confirmed by the STEM-EDS maps of the NCs derived at 250 °C (Figure S11). This observation was further corroborated by the XRD pattern of the aliquot at 250 °C, which displayed the characteristic peaks from both CuBi_5_S_8_ and Cu_2.94_Bi_4.8_S_9_ (Figures S3, S29, and S30).

### Zn^2+^ Diffusion and Nanorod Elongation

**Figure 4:**
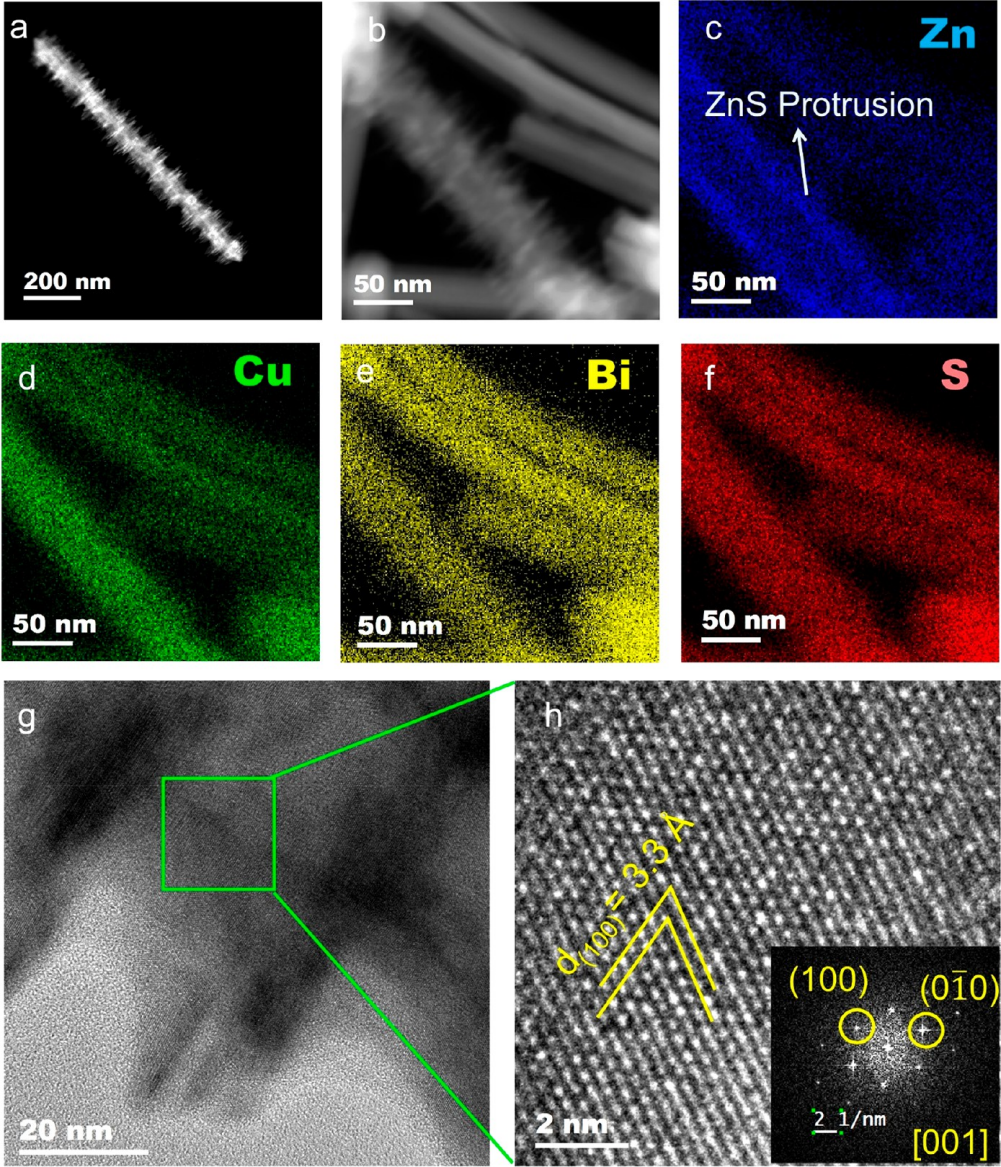
(a, b) ADF-STEM micrographs of a NR with ZnS protrusions. EDS elemental maps for (c) Zn (Kα, magenta), (d) Cu (Kα, yellow), (e) Bi (Lα, cyan), and (f) S (Kα, red) and of the NR with ZnS protrusions along with the NRs with homogeneous Zn distribution. (g) LR-TEM and (h) HRTEM images of the ZnS protrusion with an accompanying selected area FFT pattern in the inset.

The heterostructures with transitional segments gradually transform into nanorods (NRs) after 10 min at 250 °C, and Zn ions (Zn^2+^) present in the reaction solution start to diffuse into the NRs. NR samples collected between 10 and 35 min displayed peripheral protrusions as presented in Figure 4a. Visualization of the NRs from STEM-EDS elemental mappings (Figure 4c–f) shows that the edges are significantly rich in Zn (Figure 4c), suggesting the formation of a Zn-rich shell around the NRs. This is further corroborated by the selected area EDS spectra shown in Figure S14b,c, whereas the NRs without the protrusion display a homogeneously distributed Zn concentration (Figures 4c and S15). The selected area FFT analysis of these protrusions revealed a *d*-spacing of 3.3 Å for the (100) and (001) planes, which match well with that of the hexagonal ZnS phase (Figure 4g,f and Figure S16). Hence, a ZnS shell forms around the edges of the NRs before the diffusion of Zn cations into the NRs.

### Multinary Nanorods

**Figure 5:**
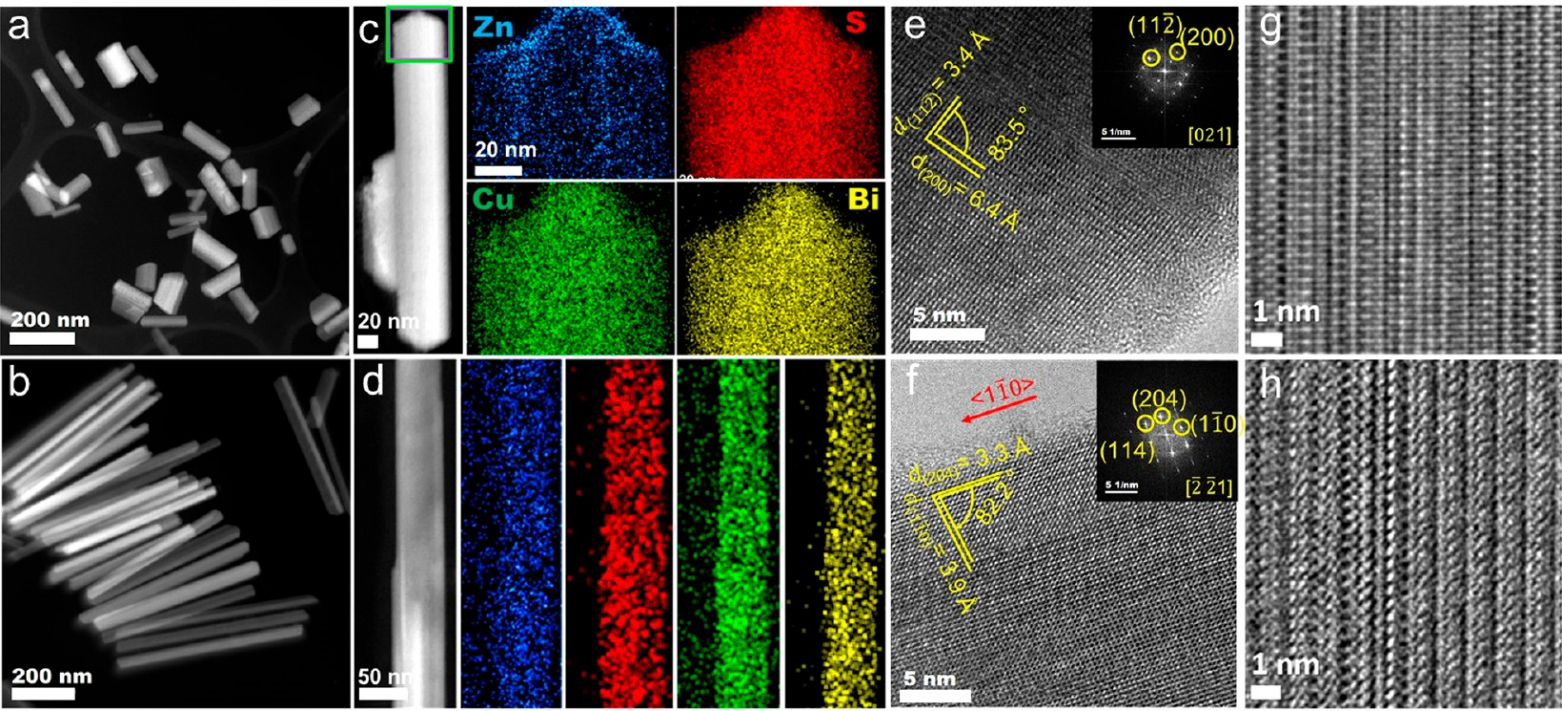
Low-resolution STEM micrographs of the (a) Zn-poor NR1 and (b) Zn-rich NR2. STEM-ADF micrographs with EDS elemental maps for Bi (yellow), Cu (Green), S (red), and Zn (blue) of (c) NR1 and (d) NR2. HRTEM of the NR1 with the corresponding FFT (inset) viewed from the [021] zone axis. (f) HRTEM of the NR2 with the corresponding FFT (inset) viewed from the [2̅2̅1] zone axis. Filtered HRTEM revealing the alternating crystal structures of (g) NR1 and (h) NR2.

The growth of the transitional segment accompanied by complete dissolution of the seed and stem segments and Zn^2+^ diffusion, lead to the formation of the Cu–Bi–Zn–S NRs after 35 min at 250 °C as shown in Figure 5a,b. The NRs formed at a ZnCl_2_ to BiCl_3_ molar ratio of 0.8:1 are shorter (Figure 5a) with an aspect ratio of ∼2.9, hereafter referred to as NR1(Figure S14a–d), exhibiting a low Zn concentration (<5%) in the SEM-EDS and ICP-OES analysis (Figure S13). At 1:1 molar ratio of ZnCl_2_ and BiCl_3_ the NRs (denoted as NR2) are longer (Figure 5b) with a higher aspect ratio of ∼10.7 (Figure S14e–h). The presence of all of the elements (Bi, Cu, S, and Zn) was confirmed by STEM-ADF EDS elemental mappings (Figure 5c,d), XPS analysis (Figure S13a), SEM-EDS (Figure S13b), and ICP-OES (Figure S13c). The XPS analysis confirmed the presence of Bi^3+^, Bi^5+^, Zn^2+^, and Cu^+^ for both crystal phases. SEM-EDS and ICP-OES analyses of NR1 and NR2 show a NR stoichiometry of Bi_5.4–4.8_Cu_3.6–4.1_Zn_0.2–0.9_S_9_ and Bi_9.2–9.0_Cu_7.7–7.2_Zn_8.5–9.1_S_22_, respectively. The growth direction of both types of NRs is along the ⟨11̅0⟩ direction as confirmed through HRTEM analysis (Figure 5e–h and Figure S19). For NR1, the *d*-spacings of the (200), (600), (800), and (11̅2̅) lattice planes closely matched with the monoclinic Cu_2.94_Bi_4.8_S_9_ phase (Figure 5e and S19 a-c). The NR2 displays *d*-spacings closely matching the (112̅), (204), (11̅0), and (600) lattice planes of the monoclinic cuprobismutite structural type (Figures 5f and S19d,e). Rietveld refinement of the XRD data from both NR samples reflects phase similarity between NR1 with the monoclinic Cu_2.94_Bi_4.8_S_9_ phase and NR2 with the monoclinic cuprobismutite structural type (Figures S17 and S18).

### Nanorod Formation Mechanism

**Figure 6:**
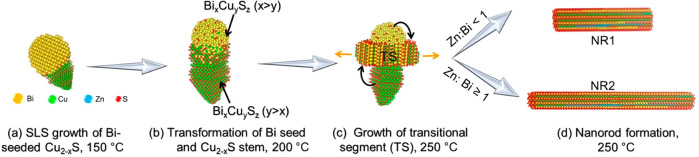
Schematic illustration depicting the evolution sequence of the Cu–Bi–Zn–S NRs. (a) Solution–liquid–solid (SLS) growth of Bi-seeded Cu_2–*x*_S stem. (b) Cation diffusion into the stem from the transformed Bi-rich Bi_*x*_Cu_*y*_S_*z*_ seed. (c) NR formation initiated with a transitional segment being materialized at the heterointerface. (d) Zn^2+^ diffusion-based Bi concentration in the reaction solution to form Cu–Bi–Zn-S NRs with different aspect ratios.

The NR evolution mechanism can be described as shown in Figure 6 and Figure S1. The growth begins with Bi-seeded Cu_2–*x*_S heterostructure formation (Figure 6a). The formation of Bi NPs can be attributed to the tendency of oleylamine (OLA) to reduce bismuth chloride by forming the Bi–OLA complex (eq 1).^39−41^ The presence of proton accepting anions such as acetylacetonate [from Cu(acac)_2_], or Cl^–^ in the coordination sphere can deprotonate the amine, resulting in transient metal–amido complex with formation of HCl or acetylacetone forms as side products. The addition of thiol (R–SH, R = alkyl chain), a mild reducing agent, can induce the reduction of the Bi–OLA complex to form Bi NPs where the thiols form disulfides (R–S–S–R) as a product as in the proposed reactions.^42,43^
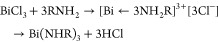
1
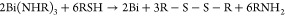
2The *in situ*-generated Bi NPs catalyze the SLS growth of Cu_2–*x*_S NCs, leading to the formation of Bi-seeded Cu_2–*x*_S heterostructure NCs (Figure 6a). In catalyst assisted growth, the metal seeds (*e.g.*, Sn and Bi) with low surface tension reduce the surface energy of the growth phase by forming a thin layer around the sidewalls of the stem, known as a wetting layer.^44,45^ Similarly, the presence of the Bi wetting layer corroborates the presence of elemental Bi in the liquid phase at the solution–liquid interface during the initial stages of heteronucleation. The small size (<7 nm) of the Bi NPs (Figure S2a–d) in the early stages of growth is responsible for such melting point depression of metallic bismuth below 150 °C.^27,46^ Thus, the initial heteronucleation process can be specified as an SLS growth of Cu_2–*x*_S from Bi seeds as depicted in Figure S2i.

The thermolysis of alkanethiol produces a sulfide source to sulfurize the metallic seed and the simultaneous incorporation of Cu^+^ transforms the metallic Bi to the CuBi_5_S_8_ ternary phase. The high ionic mobility of Cu^+^ in the djurleite lattice above 103 °C creates available sites for foreign cation incorporation into the Cu_2–*x*_S stem.^21^ The transformed CuBi_5_S_8_ seed enables the diffusion of Bi cations into the ion-conducting Cu_2–*x*_S stem (Figure 6b). With the increased temperature above 170 °C, more Bi cations diffuse into the Cu_2–*x*_S to form a ternary monoclinic Bi_*x*_Cu_*y*_S_*z*_ phase which is close with the monoclinic Cu_2.94_Bi_4.8_S_9_ phase. Alloying of seed and stem elements form a transitional segment at the heterointerface (Figure 6c). The Cu-rich and Bi-rich Bi_*x*_Cu_*y*_S_*z*_ segments, stem, and seed, respectively, act as reservoirs of cationic entities for the growth of the transitional segment along the ⟨11̅0⟩ direction forming a trisegmental heterostructure. The seed transfers material to the transitional segment as the growth progresses and is consumed upon completion of the rod formation. In the trisegmental heterostructure, stem and transitional segments are very similar in crystal structure with different elemental compositions (Figure 6c). Therefore, it can be proposed that material is transferred from the stem to the transitional segment and dissolves as the growth progresses. Deformity of the stem shape of the heterostructure derived from the aliquot at 230 °C (Figure S11) suggests such recrystallization of the stem segment. The smaller NCs (Figure S8) also dissolve into the transitional growth segment as a form of ripening toward NR formation.

It is understandable from the marginal Zn concentration in the heterostructures that the Zn incorporation occurs at the final stages of the nanorod formation where it affects the axial elongation of the nanorods (Figure 6d). It is worth noting that the Zn plays no role in the heterostructure evolution and will come into effect only after 230 °C as suggested in XPS analysis of the aliquot in Figure S32. A reactive precursor of Zn in the presence of excess S-source will prefer heteroepitaxial growth of ZnS during cation exchange processes of ternary metal copper chalcogenide above 230 °C.^47^ Reduction in the precursor reactivity will allow cationic diffusion besides shell formation.^48^ According to Pearson’s hard–soft acid base (HSAB) theory, Zn^2+^ and Cl^–^ are borderline Lewis acid and bases, respectively;^49^ thus, ZnCl_2_ being a less reactive precursor initially forms a ZnS shell around the Cu_2.94_Bi_4.8_S_9_ NRs. With time at 250 °C, Zn diffuses homogeneously within the NR to form quaternary Cu–Bi–Zn–S-based NRs depending upon the relative Bi concentration in the reaction mixture. In view of HSAB theory, both Bi^3+^ and Zn^2+^ are borderline Lewis acids, whereas Bi^5+^ is a stronger Lewis acid.^50,51^ But tri-/pentavalent cations, unlike bivalent cations, can satisfy the charge and coordination balance at the tetrahedral sites involving monovalent Cu^+^.^21^ Therefore, the incorporation of Bi^3+^ or Bi^5+^ is preferred to Zn^2+^. As a result, the system containing a higher amount of Bi source enables low Zn concentration by occupying the vacant sites in the crystal lattice leading to shorter NRs, whereas higher Zn concentration results in NR elongation. For example, the shorter NRs (NR1) are formed when the molar ratio of ZnCl_2_ to BiCl_3_ is 0.8:1 or above. In contrast, when the molar ratio of ZnCl_2_ to BiCl_3_ is 1 or lower, longer NRs (NR2) were formed. For the same reason, a longer growth time that enabled higher Zn incorporation resulted in elongated NRs (Figure S14).

### Thermoelectric Properties of the Nanorods

**Figure 7:**
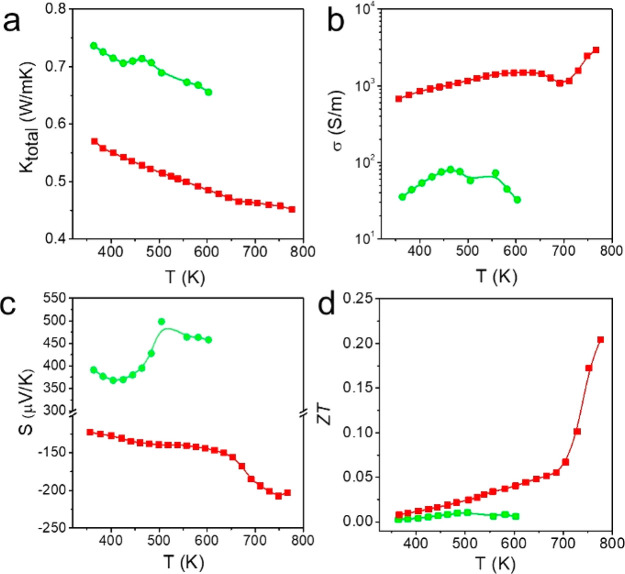
(a) Thermal conductivity, *k*, (b) electrical conductivity, σ, (c) Seebeck coefficient, *S*, and (d) thermoelectric figure of merit, ZT, of NR1 denoted by red squares and NR2 denoted by green spheres.

The complex structure and multinary composition of the produced heterostructures were expected to result in low thermal conductivities. Thus, the thermoelectric (TE) properties of NR1 and NR2 were investigated. The thermal conductivity (κ), electrical conductivity (σ), Seebeck coefficient (*S*), and calculated figure of merit (ZT) for both NR1 and NR2 are presented in Figure 7a–d. NR1 was indeed characterized by very low thermal conductivity ranging from 0.58 to 0.45 W/mK in the temperature range of 364 and 775 K. NR2 also displayed a low thermal conductivity in the range from 0.73 to 0.65 W/mK in the temperature range of 364–605 K. The presence of Bi^3+^ and the complex low symmetry crystal structure helped stereochemical activity of Bi 6S^2^ electrons, which results in effective phonon scattering. In addition, the relatively large primitive cell of the NCs could contribute to reducing the acoustic phonon population. Large cell parameters are responsible for the reduced volume of the first Brillouin space which in turn translates the high-frequency acoustic vibrations modes to weak optical modes with a nominal contribution to the lattice thermal conductivity. The cumulative effect from both parameters explained the intrinsically low thermal conductivity. The high Zn substitution in NR2 resulted in a reduced charge carrier concentration and higher effective mass. As a result, NR2 displays a low σ and high S. The higher band gap of 1.95 eV for NR2 compared to 1.38 eV for NR1 also supports the above observations (Figure S20). The positive value of S for NR2 indicates a p-type conductivity, whereas the negative value of S for NR1 indicates an n-type behavior. The low σ of the NR2 resulted in a low power factor (Figure S21a) and ZT, whereas the relatively high σ of the NR1 and significantly low κ result in an enhanced ZT of 0.21 at 775 K for the NR1.

## Conclusion

Our study reveals that a metallic seed for metal chalcogenide nanocrystals can be subsequently incorporated into the nanocrystal in tandem with additional metal ion incorporation to achieve complex multinary semiconductor NRs. Specifically, in this synthesis, *in situ*-generated Bi NPs catalyzes the SLS growth of Cu_2–*x*_S NCs. At a relatively higher reaction temperature (>190 °C), the Bi seed and Cu_2–*x*_S stem transform into monoclinic Bi_*x*_Cu_*y*_S_*z*_ phases, which dissolve into the transitional growth segment to form alloyed NRs. The Zn incorporation occurs only at higher temperatures (>230 °C), and Zn has no role to play during the initial heteronucleation. Finally, the diffusion of in-solution Zn^2+^ forms ZnS protrusions on the NRs, and subsequent Zn^2+^ diffusion from the protrusions materializes the Cu–Bi–Zn–S composition. At a relatively low Bi concentration, a longer growth time at 250 °C enables high incorporation of Zn^2+^, leading to axial elongation. Furthermore, the TE properties of the Cu–Bi–Zn–S NRs were studied where the NRs exhibited n-and p-type transport properties based on the Zn concentration. The n-type and p-type NRs both display low thermal conductivity values of 0.58 to 0.45 W/mK (364 to 775 K) and 0.73 to 0.65 W/mK (364 to 605 K), respectively. The present study epitomizes the link between conventional colloidal growth mechanisms and seeded growth mechanisms for facile pathways to synthesize alloyed multielement NCs.

## Experimental Section

### Chemicals

Copper acetylacetonate [Cu(acac)_2_, 97%, Lot No. STBD3281 V], bismuth chloride (BiCl_3_, ≥98%, Lot No. MKBV5130 V), zinc chloride (ZnCl_2_, 99.99%, Lot No. MKBF6398 V), trioctyl phosphine (TOPO, 99%, Lot No. MKCF0650), *tert*-dodecylmercaptan (*t*-DDT, 98.5%, Lot No. STBH4978), 1-dodecanethiol (1-DDT, ≥98%, Lot No. STBF43147 V), oleyl amine (OLA, 70%, Lot No. STBJ0354), and 1-octadecence (ODE, 90%, Lot No. NKBL4740 V) were purchased from Sigma-Aldrich. Toluene (Tol), methanol (MeOH), and isopropanol (IPA) were purchased from Lennox, Ireland. The chemicals were used as received without any further purification.

### Cu–Bi–Zn–S Nanocrystal Synthesis

In a typical synthesis, 130.9 mg (0.5 mmol) of Cu(acac)_2_, 157.7 mg (0.5 mmol) of BiCl_3_, 68.2 mg (0.5 mmol) of ZnCl_2_, and 676.5 mg of TOPO were mixed with a 2 mL of OLA and 8 mL of ODE solvent mixture in a three-neck round-bottom flask (RBF), and the reaction mixture was evacuated at 50 °C for 45 min. The vacuum pressure was kept below 200 mTorr during evacuation. Afterward, the reaction mixture was heated to 250 °C under an argon atmosphere. A 1 mL aliquot of thiol mixture (0.875 mL of 1-DDT and 0.125 mL of *t*-DDT) was injected when the temperature reached 135 °C. After thiol injection the reaction mixture turned to bright orange from green and finally turned to black above 147 °C. When the temperature of the reaction vessel reached 250 °C it was allowed to proceed for another 35 min to form NR2. Afterward the heating mantle was removed to terminate the reaction by natural cooling until 80 °C. Upon reaching 80 °C, 8 mL of Tol was injected to quench the reaction. For synthesizing the Zn-poor NR1, 236.5 mg BiCl_3_ was employed keeping the other conditions same as above.

### NC Purification Procedure

The NRs synthesized and quenched with 8 mL of Tol were poured into a 50 mL centrifuge tube and a further 2 mL of IPA was added and vortexed well. After that, the NR solution was centrifuged at 5000 rpm for 3 min. The supernatant was collected with a pipet, and 5 mL of MeOH was added into the supernatant solution. The solution was centrifuged at 5000 rpm for 5 min. The pellet was collected and dispersed in 2 mL of Tol, and 8 mL of IPA was further added and vortexed to disperse the NRs well. The NR solution was again centrifuged at 5000 rpm for 5 min, and the process was repeated another time as displayed in Figure S29.

### Aliquot Study

During NC growth, 1 mL of solution from the RBF was withdrawn at a desired temperature and time after thiol injection. Two adjacent temperature windows were used in each case. For example, if the first aliquot was withdrawn at 150 °C, the second one was withdrawn at 160 °C. To ensure minimal depletion in precursor concentration, a maximum of 2 mL of reaction solution in total was withdrawn from RBF. After withdrawal, the growth was immediately quenched by ejecting into 2 mL of Tol. The NCs in 2 mL of Tol were dispersed in 2 mL of IPA and centrifuged for 5 min at 5000 rpm. Followed by another two cycles of redispersion in 2 mL of Tol and 2 mL of IPA and centrifugation at 5000 rpm for 3 min.

### Electron Microscopy

For transmission electron microscopy analysis the NCs were dispersed in Tol and drop-cast on continuous carbon-coated 200 mesh nickel grids. Low-resolution and high-resolution TEM and dark-field scanning transmission electron microscopy (DFSTEM) were conducted by using a 200 kV JEOL JEM-2100F field emission microscope, equipped with a Gatan Ultra scan CCD camera and EDAX Genesis energy dispersive X-ray spectroscopy detector. Aberration-corrected TEM and scanning transmission electron microscopy analysis was carried out using a Thermo-Fisher Scientific double-tilt STEM holder in the Thermo-Fisher Scientific FEI double-aberration-corrected monochromatic Titan Themis Z. The microscope was operated at 300 kV. The imaging mode used was STEM annular dark-field mode at 91, 146, 230, and 460 mm camera lengths with a 50 μm C2 aperture depending on the imaging mode required, *i.e.*, high-, medium-, or low-angle annular dark field. EDS mapping was done using a Bruker Super X detector. All Titan STEM and EDS data processing was done using Thermo-Fisher Scientific Velox Software. TEM imaging was done using a Gatan OneView camera. All final images are an average from a series stack of low-dose images to avoid beam damage and data as processed using GMS3 software. Scanning electron microscopy of the NC drop-cast film on a stainless-steel substrate was performed on a Hitachi SU-70 system at 10 kV accelerating voltage.

### HRTEM Data Processing

For analyzing the HRTEM data, interplanar distances and the exact orientation of particle were determined from the selected area FFT analysis. On the preprocessing stage, all raw files were automatically processed with the use of free pygwy library (part of Gwyddion software^52^). For each frame, the image and its 2D FFT were prepared. To simplify the analysis of interplanar distances observed on each frame, the angular integration of 2D FFT patterns was performed as well; this operation allows one to directly confirm the appearance of all periodic features, observable on the image, and easily find corresponding periods, *i.e.*, interplanar distances in reciprocal coordinates. To improve the signal-to-noise ratio, integration has been performed as a calculation of maximum value for each value of Fourier frequency on the PSDF plot (see http://gwyddion.net/documentation/user-guide-en/statistical-analysis.html and https://github.com/LebedevV/Useful-scripts/blob/master/gwy_convert.py for details). These results were employed for the verification of phase composition of the heterostructures and nanorods by comparison with the PXRD data and corresponding databases (COD, PDF4+, and PDF numbers are mentioned with the XRD patterns). Example of data calculated one may find in Figures S7a (inset) and S17. For more detailed analysis, cropped images with the only one crystalline particle were analyzed in a similar way, but with the manual processing of 2D FFT data in Gatan digital micrograph software. As far as periodical fringes on HR-TEM images corresponding to sets of crystal planes and 2D FFT transform of square images preserves angles, one may use approaches from ED or SC-XRD for indexing and analysis of these FFT patterns, namely, for the known phase, the exact orientation can be determined (Figures S4 and S5) and for the unknown phase a number of restraints on symmetry and lattice parameters can be obtained. These restraints, in combination with the chemical analysis results, PXRD data, Kikuchi line observations, crystallographic databases, and literature, allowed us to confirm the exact structure type of each observed crystalline phase.

### X-ray Diffraction Analysis

XRD of drop-cast films of the NCs on the flat surface of silicon Zero background was conducted using a PANalytical Empyrean instrument equipped with a Cu Kα radiation source (λ = 1.5418 Å) and a 1D X’celerator strip detector with the diffractometer operating at 40 kV and 40 mA.

For all XRD patterns obtained, phase analysis with a free COD database^53^ and PDF4+ database with the High Score has been performed. Exact structures of Bi_*x*_Cu_*y*_S_*z*_ were assigned on the basis of the restraints from HRTEM data information from the literature^54^ and fit quality of refinement.

Therefore, all of the PXRD patterns were analyzed by Rietveld method in Jana2006 software. In the case of aliquots, only lattice and profile parameters were refined; structures were used exactly as described in the corresponding CIF files. However, for the nanorod, atomic coordinates were refined, and the possible Bi to Zn substitutions in positions Bi1 and Bi3 for Cu_2.94_Bi_4.8_S_9_ and Bi2 and Bi4 for cubrobismuthite in addition to Bi1 and Bi3 have been considered.

### UV–Vis Measurements

UV–vis–near-IR absorbance spectra were collected on a Cary 5000 UV–vis–near-IR spectrophotometer. Samples were dispersed in toluene in quartz cuvette with 1 cm path length, and the spectra were collected in a double-beam transmission mode with a lamp changeover at 800 nm.

### Thermoelectric Properties

The Seebeck coefficient and resistivity were simultaneously measured under helium atmosphere in a LSR-3 Linseis system. All samples were tested for at least three heating and cooling cycles. Considering the system and measurement accuracy and measurement accuracy, we estimated the measurement error of conductivity and Seebeck coefficient to be about 4%. Thermal conductivities were obtained by multiplying the thermal diffusivity (λ), the constant pressure heat capacity (*C*_*p*_), and the density of the material (ρ): κ_total_ = λ*C*_*p*_ρ. Thermal diffusivities were measured by a Xenon Flash Apparatus XFA 600 and a Laser Flash Analyzer LFA 1000, Linseis, which have an estimated error of *ca.* 5%. The heat capacity was estimated from the Dulong–Petit limit (3R law).

## Supporting Information Available

Additional data of SEM, TEM, HRTEM, STEM-EDS mapping, XRD patterns, and Rietveld refinement, size distribution histograms, UV–vis–near-IR spectra, XPS analysis, and FTIR analysis of the aliquot samples and nanorod samples (PDF)

## References

[ref1] Mantella V.; Ninova S.; Saris S.; Loiudice A.; Aschauer U.; Buonsanti R. Synthesis and Size-Dependent Optical Properties of Intermediate Band Gap Cu3VS4 Nanocrystals. Chem. Mater. 2019, 31 (2), 532–540. 10.1021/acs.chemmater.8b04610.

[ref2] Guria A. K.; Prusty G.; Chacrabarty S.; Pradhan N. Fixed Aspect Ratio Rod-to-Rod Conversion and Localized Surface Plasmon Resonance in Semiconducting I-V-VI Nanorods. Adv. Mater. 2016, 28 (3), 447–453. 10.1002/adma.201504377. 26584459

[ref3] Ghosh S.; Avellini T.; Petrelli A.; Kriegel I.; Gaspari R.; Almeida G.; Bertoni G.; Cavalli A.; Scotognella F.; Pellegrino T.; Manna L. Colloidal CuFeS2 Nanocrystals: Intermediate Fe d-Band Leads to High Photothermal Conversion Efficiency. Chem. Mater. 2016, 28 (13), 4848–4858. 10.1021/acs.chemmater.6b02192. 29033496 PMC5634747

[ref4] Singh S.; Singh A.; Palaniappan K.; Ryan K. M. Colloidal synthesis of homogeneously alloyed CdSexS1-x nanorods with compositionally tunable photoluminescence. Chem. Commun. 2013, 49 (87), 10293–10295. 10.1039/c3cc45497e. 24066355

[ref5] Singh A.; Singh S.; Levcenko S.; Unold T.; Laffir F.; Ryan K. M. Compositionally tunable photoluminescence emission in Cu2ZnSn(S(1-x)Se(x))4 nanocrystals. Angew. Chem., Int. Ed. Engl. 2013, 52 (35), 9120–4. 10.1002/anie.201302867. 23780738

[ref6] Ibanez M.; Zamani R.; LaLonde A.; Cadavid D.; Li W. H.; Shavel A.; Arbiol J.; Morante J. R.; Gorsse S.; Snyder G. J.; Cabot A. Cu2ZnGeSe4 Nanocrystals: Synthesis and Thermoelectric Properties. J. Am. Chem. Soc. 2012, 134 (9), 4060–4063. 10.1021/ja211952z. 22332903

[ref7] Ibanez M.; Cadavid D.; Zamani R.; Garcia-Castello N.; Izquierdo-Roca V.; Li W. H.; Fairbrother A.; Prades J. D.; Shavel A.; Arbiol J.; Perez-Rodriguez A.; Morante J. R.; Cabot A. Composition Control and Thermoelectric Properties of Quaternary Chalcogenide Nanocrystals: The Case of Stannite Cu2CdSnSe4. Chem. Mater. 2012, 24 (3), 562–570. 10.1021/cm2031812.

[ref8] Just J.; Coughlan C.; Singh S.; Ren H.; Müller O.; Becker P.; Unold T.; Ryan K. M. Insights into Nucleation and Growth of Colloidal Quaternary Nanocrystals by Multimodal X-ray Analysis. ACS Nano 2021, 15, 6439–6447. 10.1021/acsnano.0c08617. 33770436 PMC8291568

[ref9] Coughlan C.; Singh A.; Ryan K. M. Systematic Study into the Synthesis and Shape Development in Colloidal CuInxGa1-xS2 Nanocrystals. Chem. Mater. 2013, 25 (5), 653–661. 10.1021/cm302597x.

[ref10] Ibanez M.; Zamani R.; Li W. H.; Shavel A.; Arbiol J.; Morante J. R.; Cabot A. Extending the Nanocrystal Synthesis Control to Quaternary Compositions. Cryst. Growth Des. 2012, 12 (3), 1085–1090. 10.1021/cg201709c.

[ref11] Li J.; Bloemen M.; Parisi J.; Kolny-Olesiak J. Role of copper sulfide seeds in the growth process of CuInS2 nanorods and networks. ACS Appl. Mater. Interfaces 2014, 6 (22), 20535–43. 10.1021/am5061454. 25347208

[ref12] Connor S. T.; Hsu C. M.; Weil B. D.; Aloni S.; Cui Y. Phase Transformation of Biphasic Cu2S-CuInS2 to Monophasic CuInS2 Nanorods. J. Am. Chem. Soc. 2009, 131 (13), 4962–4966. 10.1021/ja809901u. 19281233

[ref13] Liu Y.; Lim C. K.; Fu Z.; Yin D. Q.; Swihart M. T. Can the Morphology of Biconcave Metal Sulfide Nanoplatelets Be Preserved during Cation Exchange?. Chem. Mater. 2019, 31 (15), 5706–5712. 10.1021/acs.chemmater.9b01686.

[ref14] Liu Y.; Liu M. X.; Swihart M. T. Shape Evolution of Biconcave Djurleite Cu1.94S Nanoplatelets Produced from CuInS2 Nanoplatelets by Cation Exchange. J. Am. Chem. Soc. 2017, 139 (51), 18598–18606. 10.1021/jacs.7b09577. 29200274

[ref15] Hinterding S. O. M.; Berends A. C.; Kurttepeli M.; Moret M. E.; Meeldijk J. D.; Bals S.; van der Stam W.; de Mello Donega C. Tailoring Cu+ for Ga3+ Cation Exchange in Cu2-xS and CuInS2 Nanocrystals by Controlling the Ga Precursor Chemistry. ACS Nano 2019, 13 (11), 12880–12893. 10.1021/acsnano.9b05337. 31617701 PMC6890264

[ref16] Chen W.; Li X. J.; Wang F.; Javaid S.; Pang Y. P.; Chen J. Y.; Yin Z. Y.; Wang S. B.; Li Y. G.; Jia G. H. Nonepitaxial Gold-Tipped ZnSe Hybrid Nanorods for Efficient Photocatalytic Hydrogen Production. Small 2020, 16 (12), 1902231 10.1002/smll.201902231. 31769587

[ref17] Carbone L.; Nobile C.; De Giorgi M.; Sala F. D.; Morello G.; Pompa P.; Hytch M.; Snoeck E.; Fiore A.; Franchini I. R.; Nadasan M.; Silvestre A. F.; Chiodo L.; Kudera S.; Cingolani R.; Krahne R.; Manna L. Synthesis and micrometer-scale assembly of colloidal CdSe/CdS nanorods prepared by a seeded growth approach. Nano Lett. 2007, 7 (10), 2942–2950. 10.1021/nl0717661. 17845067

[ref18] Ben-Shahar Y.; Philbin J. P.; Scotognella F.; Ganzer L.; Cerullo G.; Rabani E.; Banin U. Charge Carrier Dynamics in Photocatalytic Hybrid Semiconductor-Metal Nanorods: Crossover from Auger Recombination to Charge Transfer. Nano Lett. 2018, 18 (8), 5211–5216. 10.1021/acs.nanolett.8b02169. 29985622

[ref19] Shcherbakov-Wu W.; Tisdale W. A. A time-domain view of charge carriers in semiconductor nanocrystal solids. Chem. Sci. 2020, 11 (20), 5157–5167. 10.1039/C9SC05925C. 34122972 PMC8159276

[ref20] Jia G. H.; Pang Y. P.; Ning J. J.; Banin U.; Ji B. T. Heavy-Metal-Free Colloidal Semiconductor Nanorods: Recent Advances and Future Perspectives. Adv. Mater. 2019, 31 (25), 1900781 10.1002/adma.201900781. 31063615

[ref21] Kapuria N.; Ghorpade U. V.; Zubair M.; Mishra M.; Singh S.; Ryan K. M. Metal chalcogenide semiconductor nanocrystals synthesized from ion-conducting seeds and their applications. J. Mater. Chem. C 2020, 8 (40), 13868–13895. 10.1039/D0TC02895A.

[ref22] Adhikari S. D.; Dutta A.; Prusty G.; Sahu P.; Pradhan N. Symmetry Break and Seeded 2D Anisotropic Growth in Ternary CuGaS2 Nanocrystals. Chem. Mater. 2017, 29 (12), 5384–5393. 10.1021/acs.chemmater.7b01775.

[ref23] Prusty G.; Guria A. K.; Mondal I.; Dutta A.; Pal U.; Pradhan N. Modulated Binary-Ternary Dual Semiconductor Heterostructures. Angew. Chem., Int. Ed. 2016, 55 (8), 2705–2708. 10.1002/anie.201509701. 26800297

[ref24] Liu M. X.; Liu Y.; Gu B. B.; Wei X. B.; Xu G. X.; Wang X. M.; Swihart M. T.; Yong K. T. Recent advances in copper sulphide-based nanoheterostructures. Chem. Soc. Rev. 2019, 48 (19), 4950–4965. 10.1039/C8CS00832A. 31528883

[ref25] Fang Y. S.; Lv K. X.; Li Z.; Kong N.; Wang S. H.; Xu A. B.; Wu Z. Y.; Jiang F. L.; Li C. R.; Ozin G. A.; He L. Solution-Liquid-Solid Growth and Catalytic Applications of Silica Nanorod Arrays. Adv. Sci. 2020, 7 (13), 2000310 10.1002/advs.202000310. PMC7341079 32670762

[ref26] Steinhagen C.; Akhavan V. A.; Goodfellow B. W.; Panthani M. G.; Harris J. T.; Holmberg V. C.; Korgel B. A. Solution-Liquid-Solid Synthesis of CuInSe2 Nanowires and Their Implementation in Photovoltaic Devices. ACS Appl. Mater. Interfaces 2011, 3 (5), 1781–1785. 10.1021/am200334d. 21452830

[ref27] Li Z.; Kornowski A.; Myalitsin A.; Mews A. Formation and Function of Bismuth Nanocatalysts for the Solution-Liquid-Solid Synthesis of CdSe Nanowires. Small 2008, 4 (10), 1698–1702. 10.1002/smll.200800858. 18780365

[ref28] Geaney H.; Mullane E.; Ryan K. M. Solution phase synthesis of silicon and germanium nanowires. J. Mater. Chem. C 2013, 1 (33), 4996–5007. 10.1039/c3tc31123f.

[ref29] Li Y.; Shao Z.-C.; Zhang C.; Yu S.-H. Catalyzed Growth for Atomic-Precision Colloidal Chalcogenide Nanowires and Heterostructures: Progress and Perspective. J. Phys. Chem. Lett. 2021, 12, 10695–10705. 10.1021/acs.jpclett.1c02358. 34709833

[ref30] Kilian S.; McCarthy K.; Stokes K.; Adegoke T. E.; Conroy M.; Amiinu I. S.; Geaney H.; Kennedy T.; Ryan K. M. Direct Growth of Si, Ge, and Si-Ge Heterostructure Nanowires Using Electroplated Zn: An Inexpensive Seeding Technique for Li-Ion Alloying Anodes. Small 2021, 17 (10), 2005443 10.1002/smll.202005443. 33475259

[ref31] Abdul Ahad S.; Kilian S.; Zubair M.; Lebedev V. A.; McNamara K.; Ryan K. M.; Kennedy T.; Geaney H. Amorphization driven Na-alloying in SixGe1-x alloy nanowires for Na-ion batteries. J. Mater. Chem. A 2021, 9 (36), 20626–20634. 10.1039/D1TA03741B.

[ref32] Imtiaz S.; Amiinu I. S.; Storan D.; Kapuria N.; Geaney H.; Kennedy T.; Ryan K. M. Dense Silicon Nanowire Networks Grown on a Stainless-Steel Fiber Cloth: A Flexible and Robust Anode for Lithium-Ion Batteries. Adv. Mater. 2021, 33, 2105917 10.1002/adma.202105917. PMC11469259 34613631

[ref33] Aminu I. S.; Geaney H.; Imtiaz S.; Adegoke T. E.; Kapuria N.; Collins G. A.; Ryan K. M. A Copper Silicide Nanofoam Current Collector for Directly Grown Si Nanowire Networks and Their Application as Lithium-Ion Anodes. Adv. Funct Mater. 2020, 30 (38), 2003278 10.1002/adfm.202003278.

[ref34] Stokes K.; Geaney H.; Flynn G.; Sheehan M.; Kennedy T.; Ryan K. M. Direct Synthesis of Alloyed Si1-xGex Nanowires for Performance-Tunable Lithium Ion Battery Anodes. ACS Nano 2017, 11 (10), 10088–10096. 10.1021/acsnano.7b04523. 28902493

[ref35] Hobbis D.; Wang H.; Martin J.; Nolas G. S. Thermal Properties of the Very Low Thermal Conductivity Ternary Chalcogenide Cu4Bi4M9 (M = S, Se). Phys. Status Solidi RRL 2020, 14 (8), 2000166 10.1002/pssr.202000166.

[ref36] Jiang Y.; Jia F.; Chen L.; Wu L. M. Cu4Bi4Se9: A Thermoelectric Symphony of Rattling, Anharmonic Lone-pair, and Structural Complexity. ACS Appl. Mater. Interfaces 2019, 11 (40), 36616–36625. 10.1021/acsami.9b11115. 31507161

[ref37] Yarema O.; Yarema M.; Moser A.; Enger O.; Wood V. Composition- and Size-Controlled I-V-VI Semiconductor Nanocrystals. Chem. Mater. 2020, 32 (5), 2078–2085. 10.1021/acs.chemmater.9b05191.

[ref38] Paul S.; Ghosh S.; Dalal B.; Chal P.; Satpati B.; De S. K. Cation Exchange Mediated Synthesis and Tuning of Bimodal Plasmon in Alloyed Ternary Cu3BiS3-xSex Nanorods. Chem. Mater. 2018, 30 (15), 5020–5031. 10.1021/acs.chemmater.8b01269.

[ref39] Xu Z. C.; Shen C. M.; Hou Y. L.; Gao H. J.; Sun S. S. Oleylamine as Both Reducing Agent and Stabilizer in a Facile Synthesis of Magnetite Nanoparticles. Chem. Mater. 2009, 21 (9), 1778–1780. 10.1021/cm802978z.

[ref40] Mourdikoudis S.; Liz-Marzan L. M. Oleylamine in Nanoparticle Synthesis. Chem. Mater. 2013, 25 (9), 1465–1476. 10.1021/cm4000476.

[ref41] Man R. W. Y.; Brown A. R. C.; Wolf M. O. Mechanism of Formation of Palladium Nanoparticles: Lewis Base Assisted, Low-Temperature Preparation of Monodisperse Nanoparticles. Angew. Chem., Int. Ed. 2012, 51 (45), 11350–11353. 10.1002/anie.201205057. 23011969

[ref42] Jain P. K.; Manthiram K.; Engel J. H.; White S. L.; Faucheaux J. A.; Alivisatos A. P. Doped Nanocrystals as Plasmonic Probes of Redox Chemistry. Angew. Chem., Int. Ed. 2013, 52 (51), 13671–13675. 10.1002/anie.201303707. 24155083

[ref43] Chen L. H.; Li G. H. Functions of 1-Dodecanethiol in the Synthesis and Post-Treatment of Copper Sulfide Nanoparticles Relevant to Their Photocatalytic Applications. ACS Appl. Nano Mater. 2018, 1 (9), 4587–4593. 10.1021/acsanm.8b00893.

[ref44] Misra S.; Yu L. W.; Chen W. H.; Roca i Cabarrocas P. Wetting Layer: The Key Player in Plasma-Assisted Silicon Nanowire Growth Mediated by Tin. J. Phys. Chem. C 2013, 117 (34), 17786–17790. 10.1021/jp403063d.

[ref45] Yu L. W.; Fortuna F.; O’Donnell B.; Patriache G.; Roca i Cabarrocas P. Stability and evolution of low-surface-tension metal catalyzed growth of silicon nanowires. Appl. Phys. Lett. 2011, 98 (12), 123113 10.1063/1.3569817.

[ref46] Liu M. L.; Wang R. Y. Size-Dependent Melting Behavior of Colloidal In, Sn, and Bi Nanocrystals. Sci. Rep. 2015, 5, 16353 10.1038/srep16353. 26573146 PMC4648084

[ref47] Lox J. F. L.; Dang Z. Y.; Dzhagan V. M.; Spittel D.; Martin-Garcia B.; Moreels I.; Zahn D. R. T.; Lesnyak V. Near-Infrared Cu-In-Se-Based Colloidal Nanocrystals via Cation Exchange. Chem. Mater. 2018, 30 (8), 2607–2617. 10.1021/acs.chemmater.7b05187.

[ref48] Lox J. F. L.; Dang Z. Y.; Le Anh M.; Hollinger E.; Lesnyak V. Colloidal Cu-Zn-In-S-Based Disk-Shaped Nanocookies. Chem. Mater. 2019, 31 (8), 2873–2883. 10.1021/acs.chemmater.9b00005.

[ref49] Fan C. M.; Regulacio M. D.; Ye C.; Lim S. H.; Lua S. K.; Xu Q. H.; Dong Z. L.; Xu A. W.; Han M. Y. Colloidal nanocrystals of orthorhombic Cu2ZnGeS4: phase-controlled synthesis, formation mechanism and photocatalytic behavior. Nanoscale 2015, 7 (7), 3247–3253. 10.1039/C4NR07012G. 25619770

[ref50] Pearson R. G. Hard and soft acids and bases, HSAB, part 1: Fundamental principles. J. Chem. Educ. 1968, 45 (9), 581 10.1021/ed045p581.

[ref51] Ramler J.; Lichtenberg C. Molecular Bismuth Cations: Assessment of Soft Lewis Acidity. Chem.—Eur. J. 2020, 26 (45), 10250–10258. 10.1002/chem.202001674. 32428329 PMC7818483

[ref52] Gwyddion; http://gwyddion.net/ (release date Feb 11, 2022).

[ref53] Altomare A.; Corriero N.; Cuocci C.; Falcicchio A.; Moliterni A.; Rizzi R. QUALX2.0: A qualitative phase analysis software using the freely available database POW_COD. J. Appl. Crystallogr. 2015, 48, 598–603. 10.1107/S1600576715002319.

[ref54] Ciobanu C. L.; Pring A.; Cook N. J. Micron- to nano-scale intergrowths among members of the cuprobismutite series and paděraite: HRTEM and microanalytical evidence. Mineral. Mag. 2004, 68, 279–300. 10.1180/0026461046820187.

