# Supplementary material for: Subsuming the Metal Seed to Transform Binary Metal Chalcogenide Nanocrystals into Multinary Compositions
Source: ACS Nano. 2022 May 20;16(6):8917–27. doi: 10.1021/acsnano.1c11144 (PMC9245353; doi:10.1021/acsnano.1c11144)
Supplement: Supplementary file 1 — nn1c11144_si_001.pdf [file nn1c11144_si_001.pdf]

Supporting information

Subsuming the Metal seed to transform Binary Metal Chalcogenide Nanocrystals into Multinary Compositions

Nilotpal Kapuria, Michele Conroy*, Vasily A Lebedev, Temilade Esther Adegoke, Yu Zhang, Ibrahim Saana Amiinu, Ursel Bangert, Andreu Cabot*, Shalini Singh*, Kevin M Ryan*

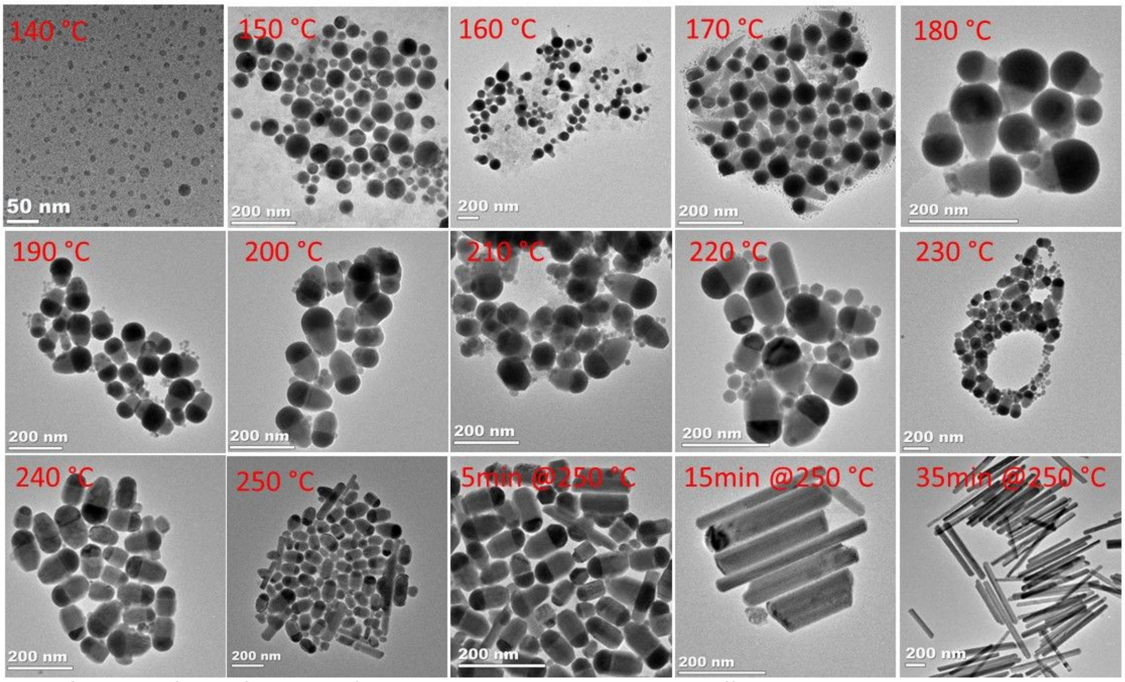

Figure S1. TEM of the NCs derived from aliquot solutions withdrawn at different temperatures displaying the Bi seed formation, Bi-$Cu_{2-x}S$ heterostructures formation, and structural evolution of the Bi-$Cu_{2-x}S$ heterostructures into Cu-Bi-Zn-S based NRs.

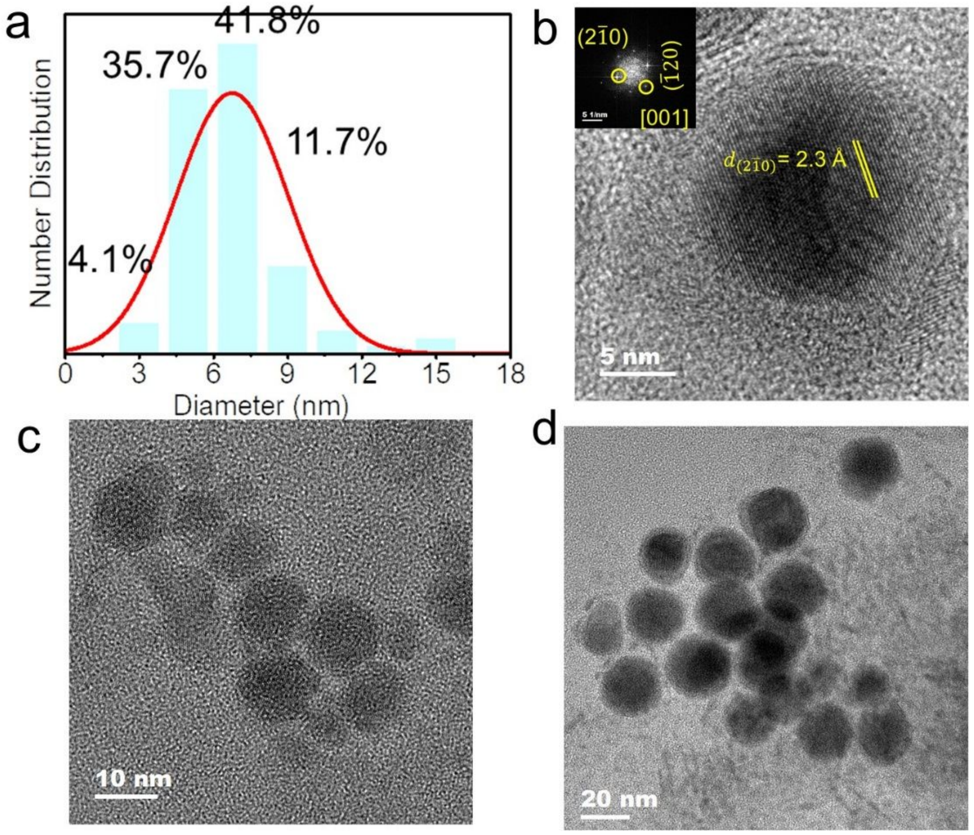

Figure S2. (a) Size distribution of the Bi NPs, (b) HRTEM of a Bi NPs collected from 140 °C aliquot; The change in size of Bi NPs due to ripening exhibited by Bi NPs from (c) 145 °C aliquot and (d) 147°C aliquot.

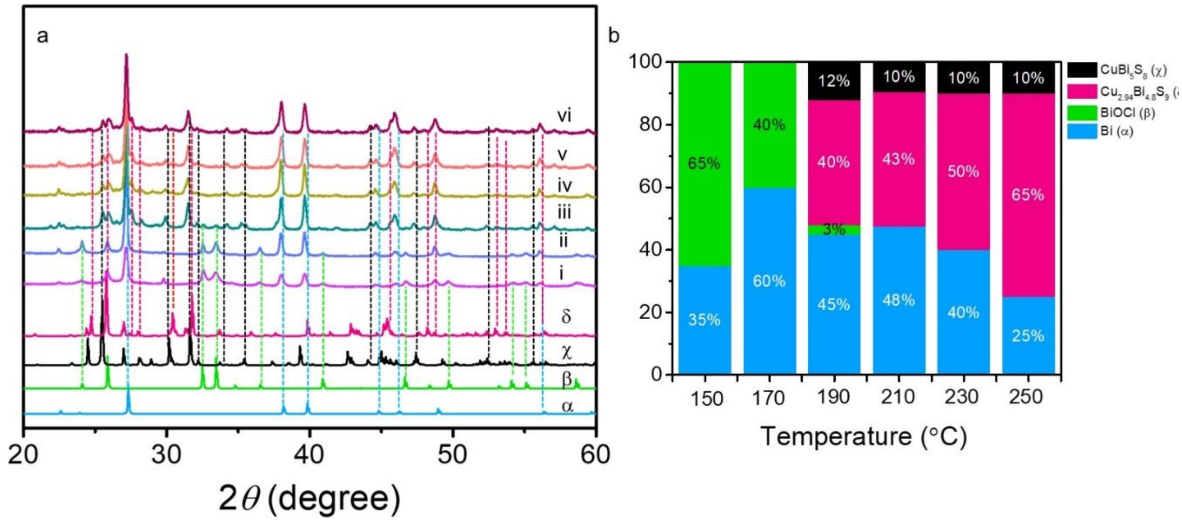

Figure S3. (a)XRD pattern of aliquots collected at (i) 150, (ii) 170, (iii) 190, (iv) 210, (v) 230, and (vi) 250 °C. The XRD pattern of rhombohedral Bi (PDF No. 04-003-1496), BiOCl (PDF No.00-006-0249), $CuBi_5S_8$ (PDF No. 01-071-2426), and $Cu_{2.94}Bi_{4.8}S_9$ (PDF No. 03-065-5469) obtained from the mentioned PDF cards marked as α, β, χ, and δ respectively (b)Percentage of the phases calculated from Rietveld refinement of the XRD patterns obtained from aliquot thin films on silicon zero background disc. From XRD analysis of aliquot 150 and 170 °C, beside Bi, presence of BiOCl is ascertained. It can be suggested at 150 and 170 °C unreacted BiCl3 is still present. Exposure of unreacted BiCl3 to air and moisture produced BiOCl (BiCl3+ H2O →2HCl+ BiOCl).

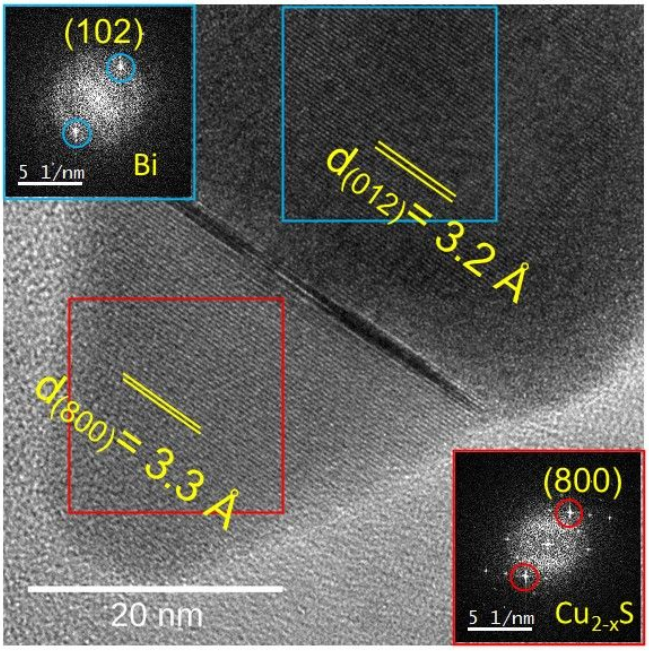

Figure S4. HRTEM image of a Bi-$Cu_{2-x}S$ heterostructure derived from the 150 °C aliquot accompanied by selected area fast Fourier transform (FFT) pattern in the insets. The $Cu_{2-x}S$ stem displays monoclinic djurleite crystal phase and the Bi seed exhibits rhombohedral phase.

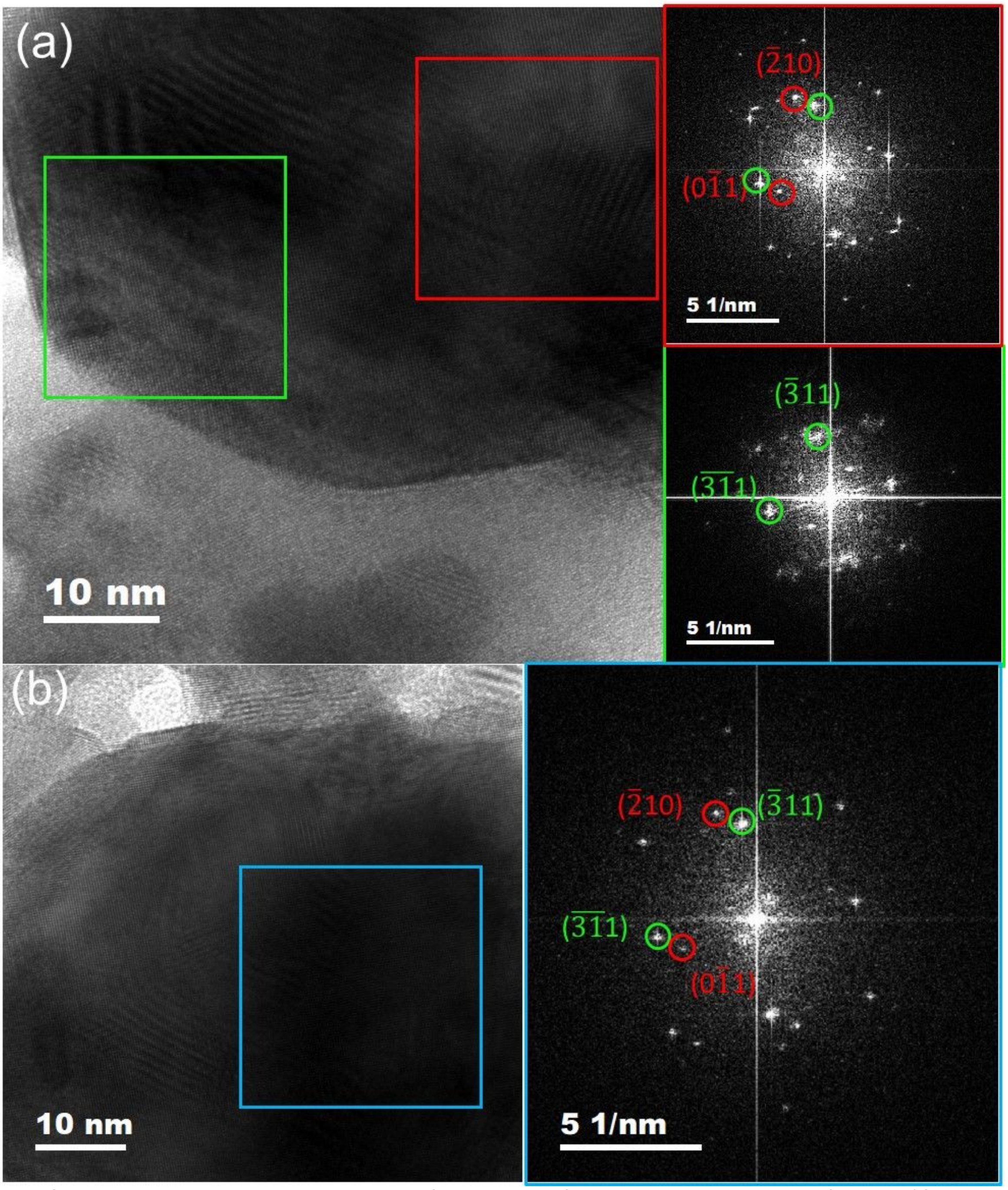

Figure S5. (a, b) HRTEM and selected area FFT of Bi rich seed of the heterostructures derived from 210 °C aliquot. The red signifies characteristic peaks from rhombohedral Bi phase and green signifies characteristic peaks from monoclinic $CuBi_5S_8$ phase.

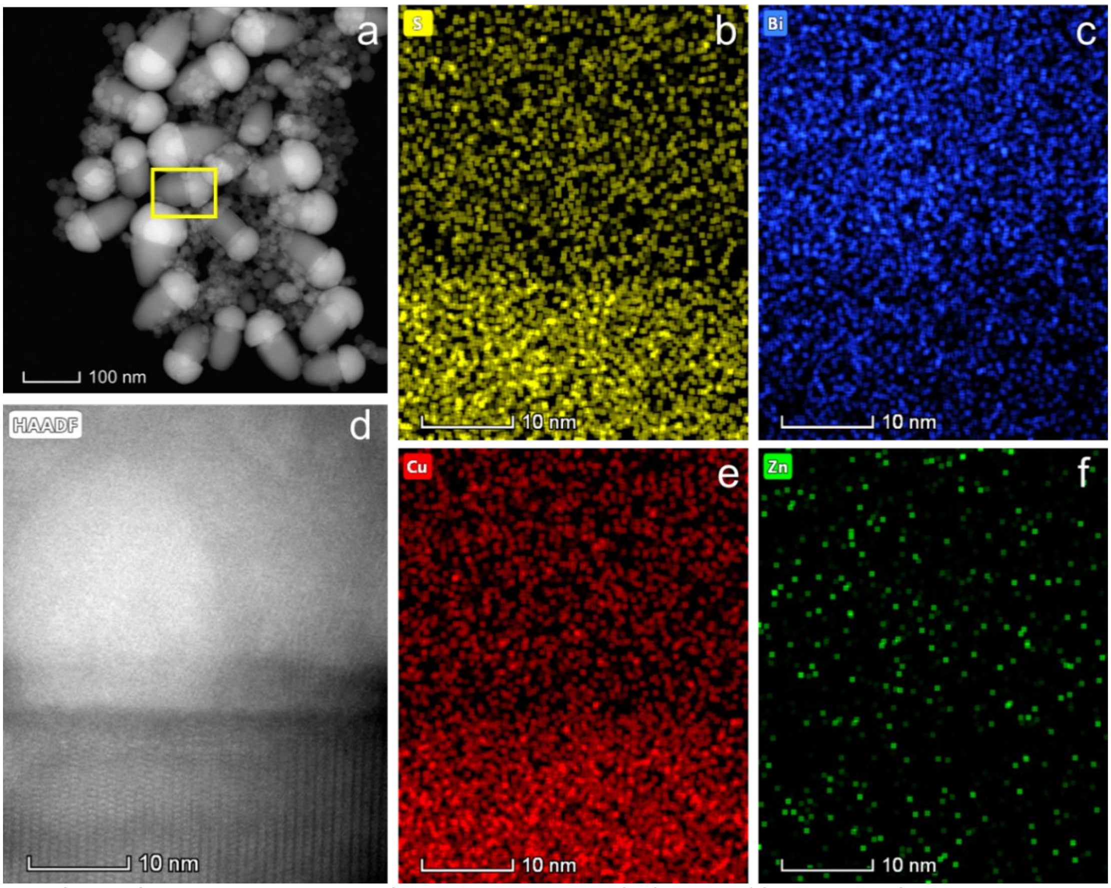

Figure S6. (a) STEM ADF micrograph of the heterostructure NCs from 210 °C aliquot, (d) STEM ADF micrograph of the hetero-interface of the NC marked by yellow square accompanying the EDS elemental maps for (b) S (yellow), (c) Bi (blue), (e) Cu (red), and (f) Zn (green).

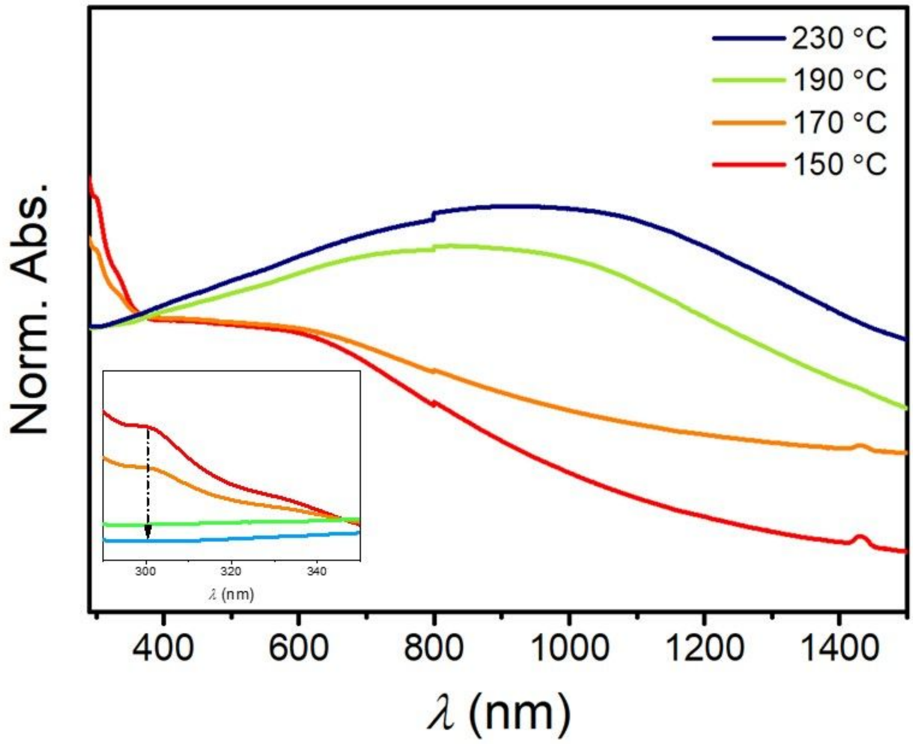

Figure S7. Absorption spectra of the aliquot solution collected at different reaction temperatures. The wavelength range of 290 to 350 nm is shown in the inset which exhibits the disappearance of excitonic feature of Bi.

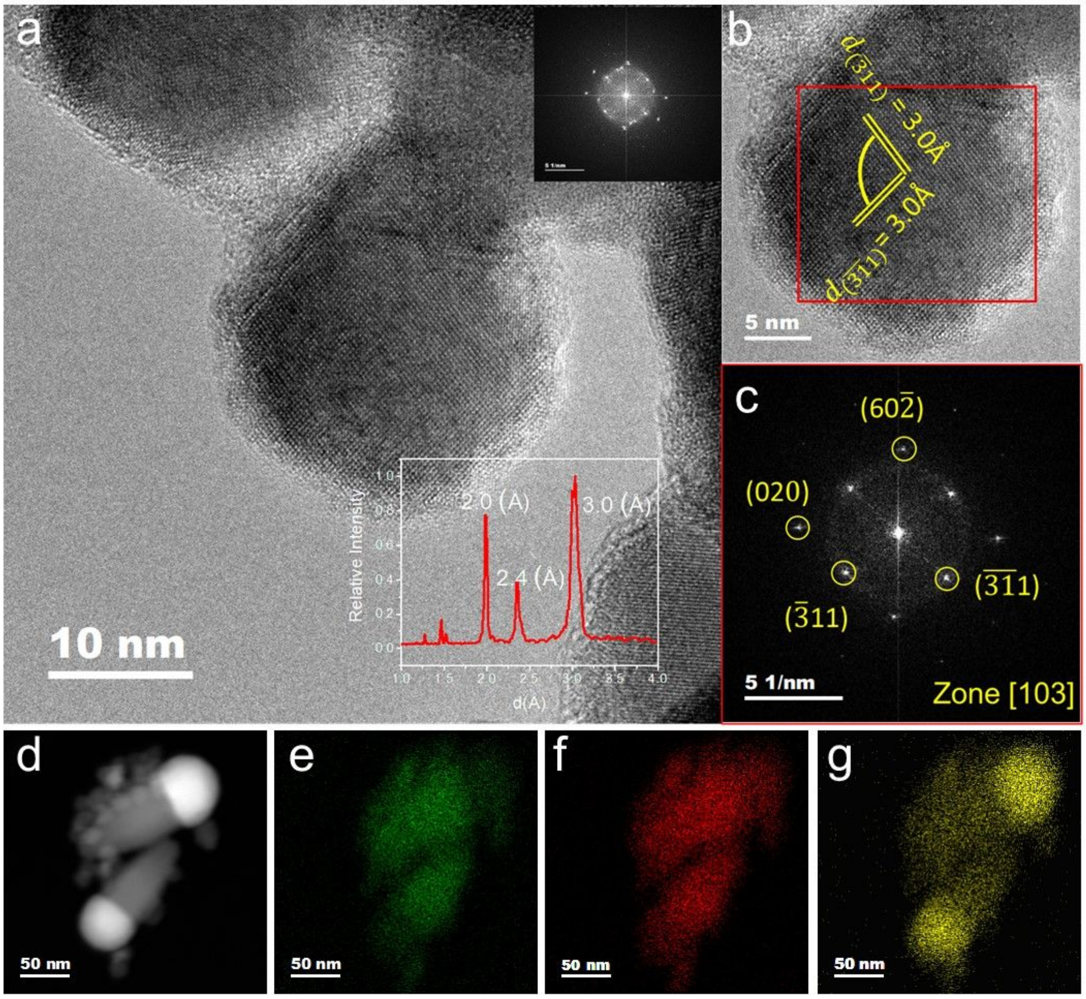

Figure S8. (a, b) HRTEM of the $CuBi_5S_8$ NCs derived from 190 °C aliquot accompanied with FFT and FFT plot; (c-f) STEM-ADF micrograph and EDS elemental mapping of the NCs derived from 190 °C aliquot. The maps for Cu(Kα), S(Kα), Bi (Lα) are displayed in green, red and yellow respectively.

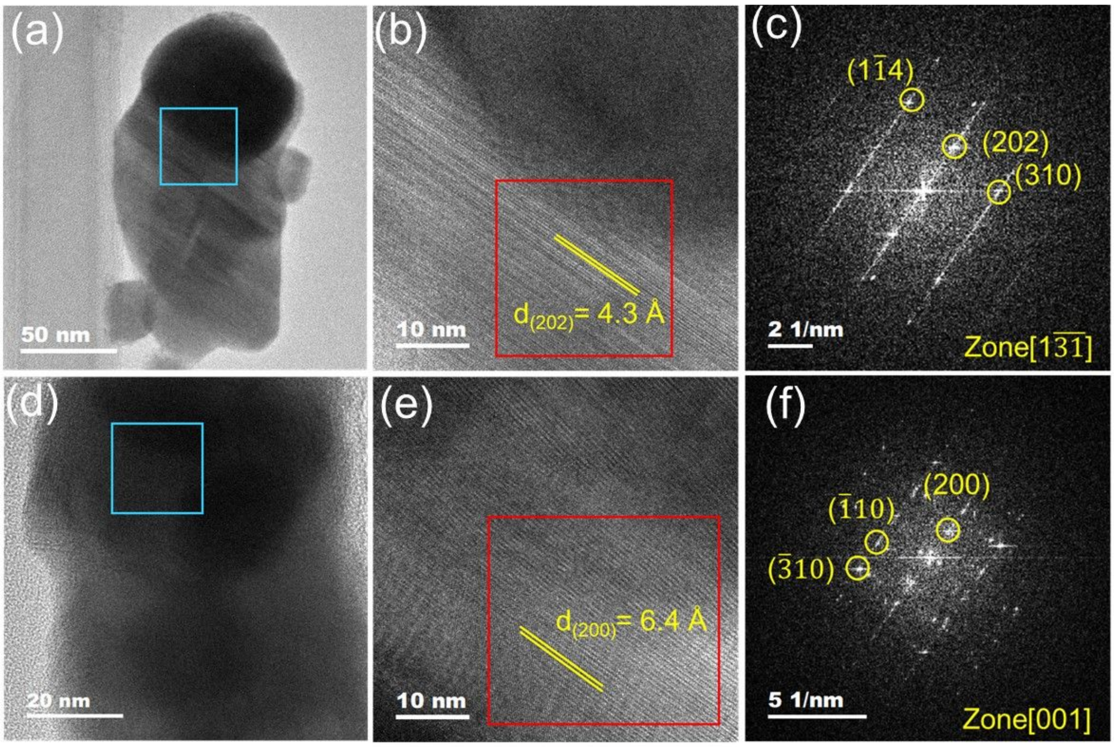

Figure S9. (a, d) HRTEM of the transitional segments of the heterostructures derived from 230 °C aliquot. (b, c, e, f) HRTEM and selected area FFTs of the transitional segments. The d-spacings for the mentioned planes matched well with that of monoclinic $Cu_{2.94}Bi_{4.8}S_9$.

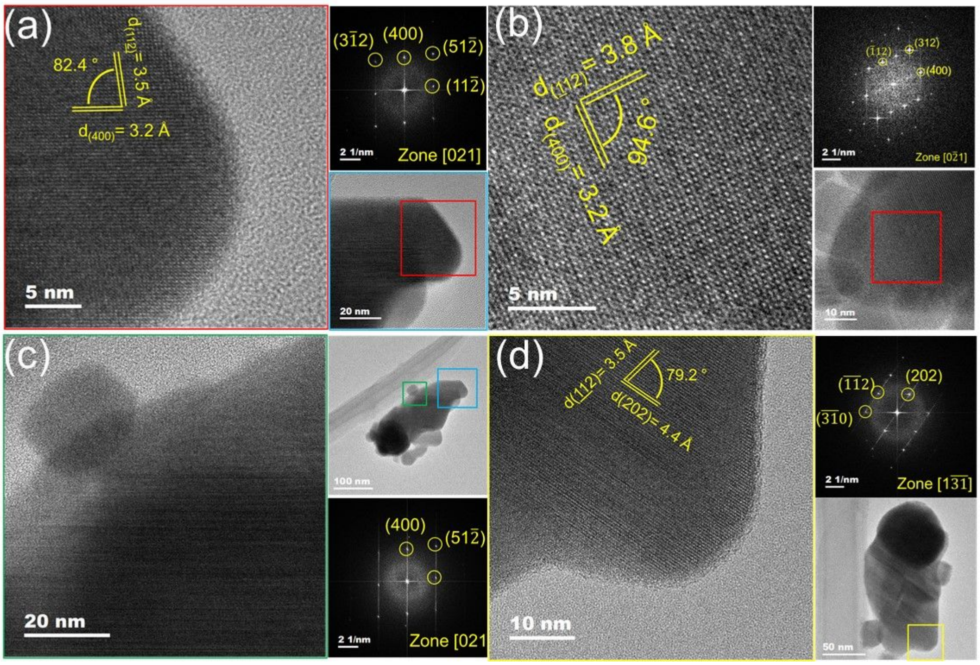

Figure S10. (a-d) HRTEM of the stem of the heterostructures derived from 230 °C aliquot accompanied with selected area FFTs.

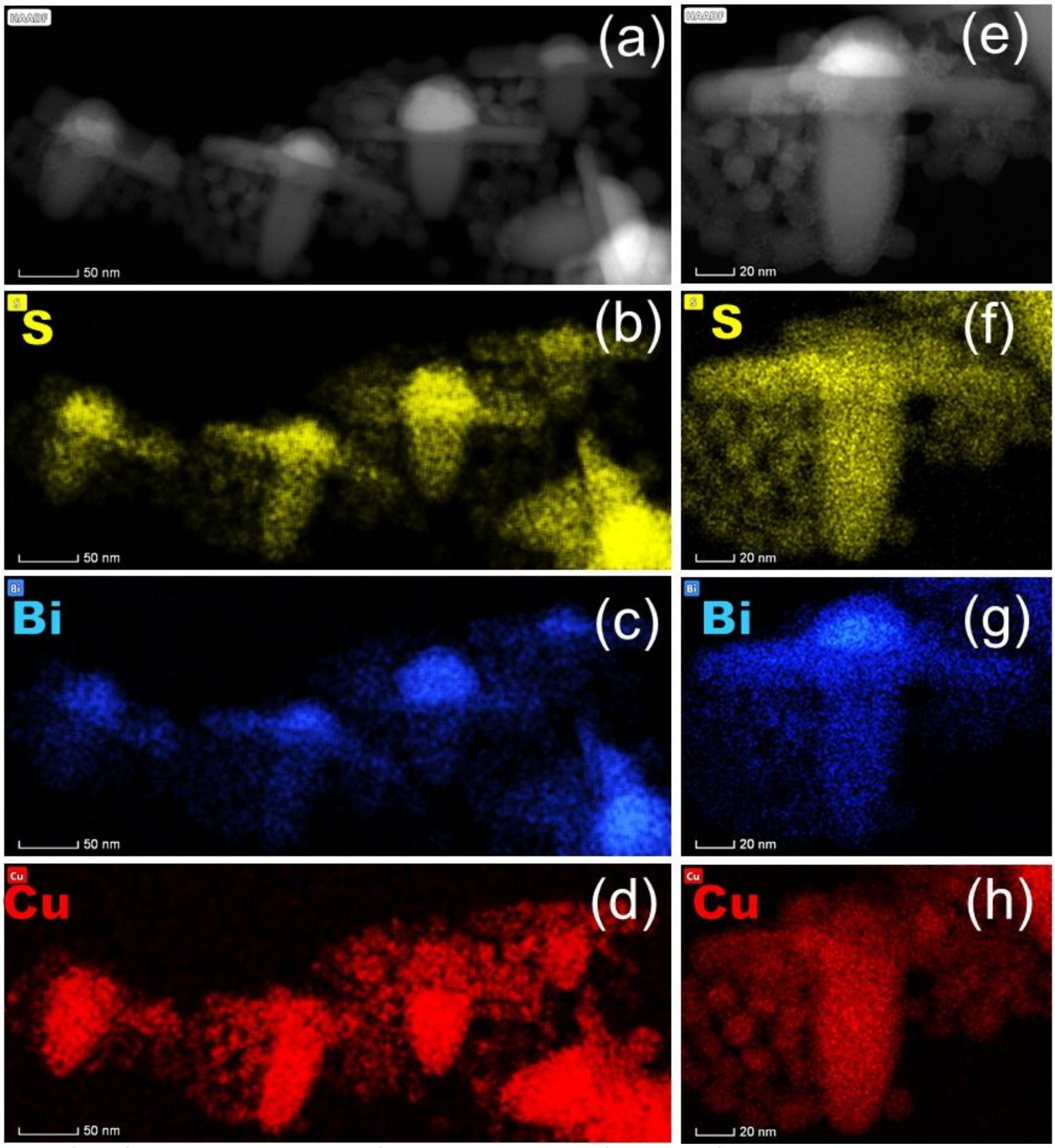

Figure S11. STEM-ADF micrographs of the heterostructure NCs derived from the aliquot collected at 250 °C accompanying with STEM-EDX element maps for S, Bi and Cu

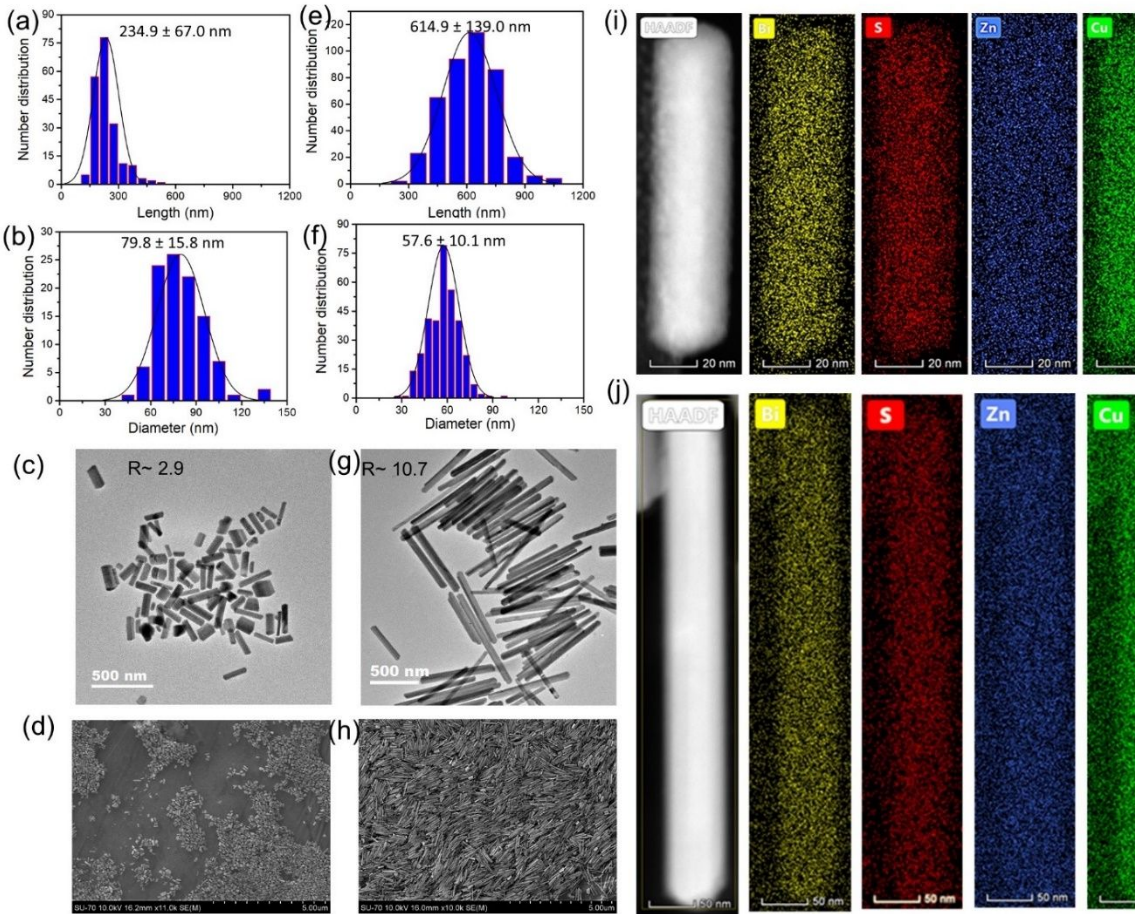

Figure S12. (a) Length and (b) diameter distribution, (c) LRTEM of NR1, and (d) SEM of NR1; (e) Length and (f) diameter distribution, (g) LRTEM, and (h) SEM of NR2; (i) STEM-ADF micrograph and elemental maps of NR1, (j) STEM-ADF micrograph and elemental maps of NR2.

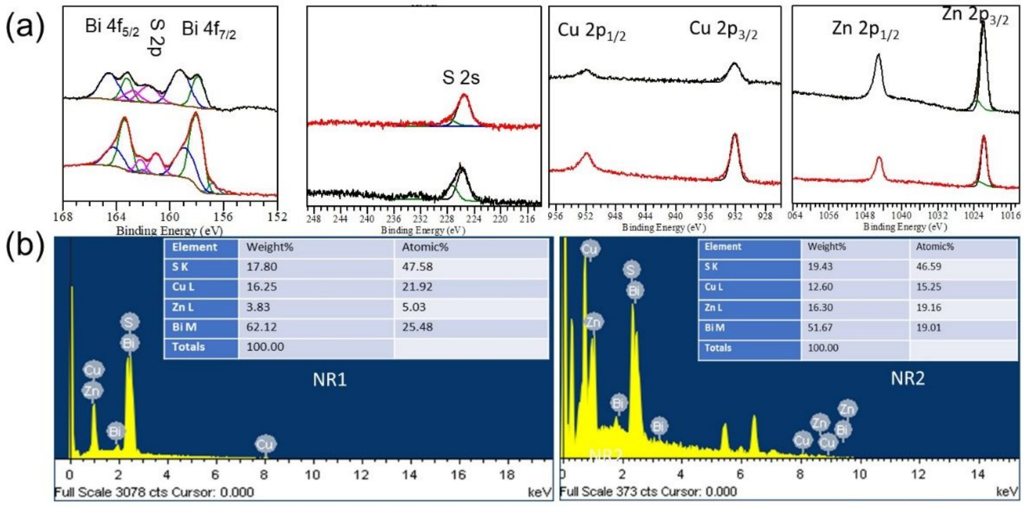

(c)

Elements	NR1	%	NR2	%
Bi (10^{-5} M)	22.1± 4.2	29.4	11.6±1.7	19.3
Cu (10^{-5} M)	15.1± 2.8	20.1	9.8±1.4	16.3
S (10^{-5} M)	37.0±6.7	49.3	27.8±4.7	46.4
Zn (10^{-5} M)	0.9± 0	01.2	10.8±1.6	17.9

Figure S13. Compositional analysis of Cu-Bi-Zn-S NRs by (a) X-ray photoelectron spectroscopy (XPS), the top spectra belong to NR1 and bottom spectra belong to NR2, (b) SEM-EDS and (c) inductively coupled plasma optical emission spectroscopy (ICP-OES).

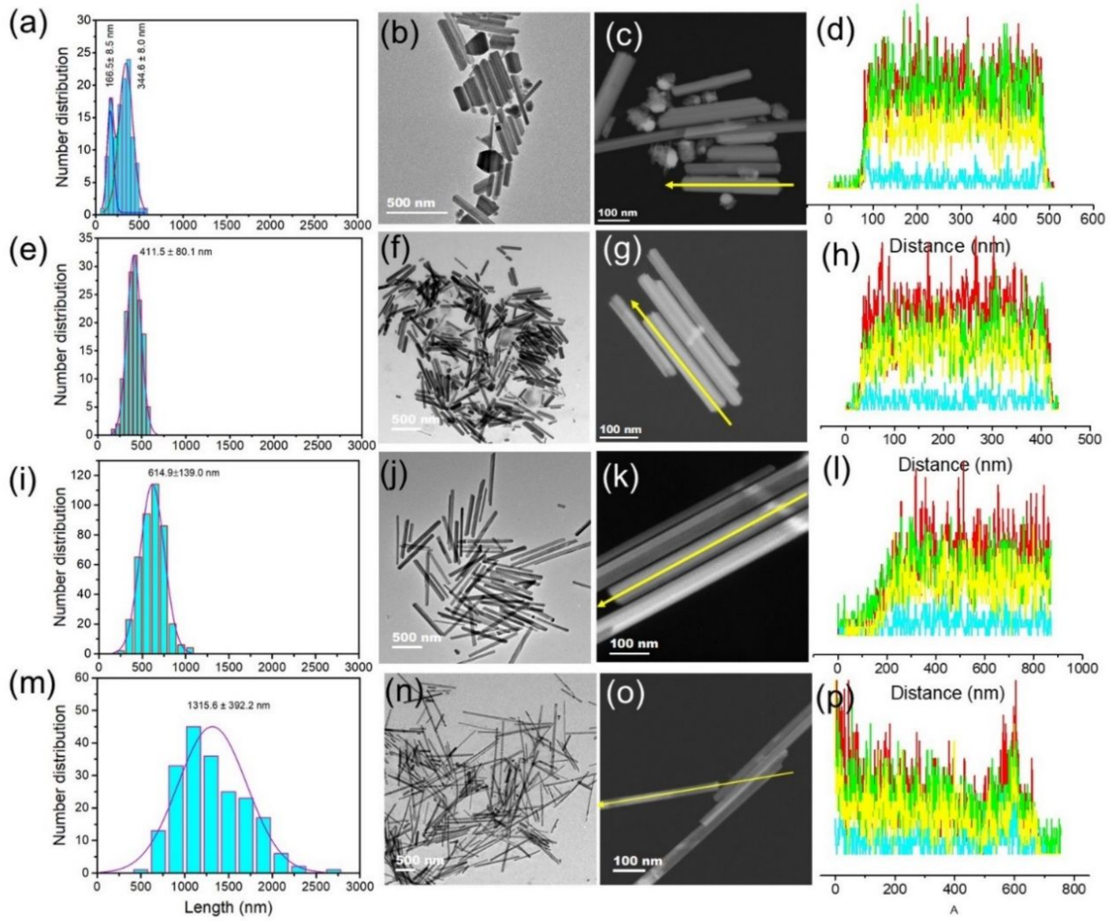

Figure S14. (a) Length distribution, (b) LRTEM, (c) STEM-ADF and (d) STEM-EDS line scan profile for the NRs formed after 15 min of growth time at 250 °C; (e) Length distribution, (f) LRTEM, (g) STEM-ADF and (h) STEM-EDS line scan profile for the NRs formed after 25 min of growth time at 250 °C; (i) Length distribution, (j) LRTEM, (k) STEM-ADF and (l) STEM-EDS line scan profile for the NRs formed after 35 min of growth time at 250 °C; (m) Length distribution, (n) LRTEM, (o) STEM-ADF and (p) STEM-EDS line scan profile for the NRs formed after 15 min of growth time at 250 °C

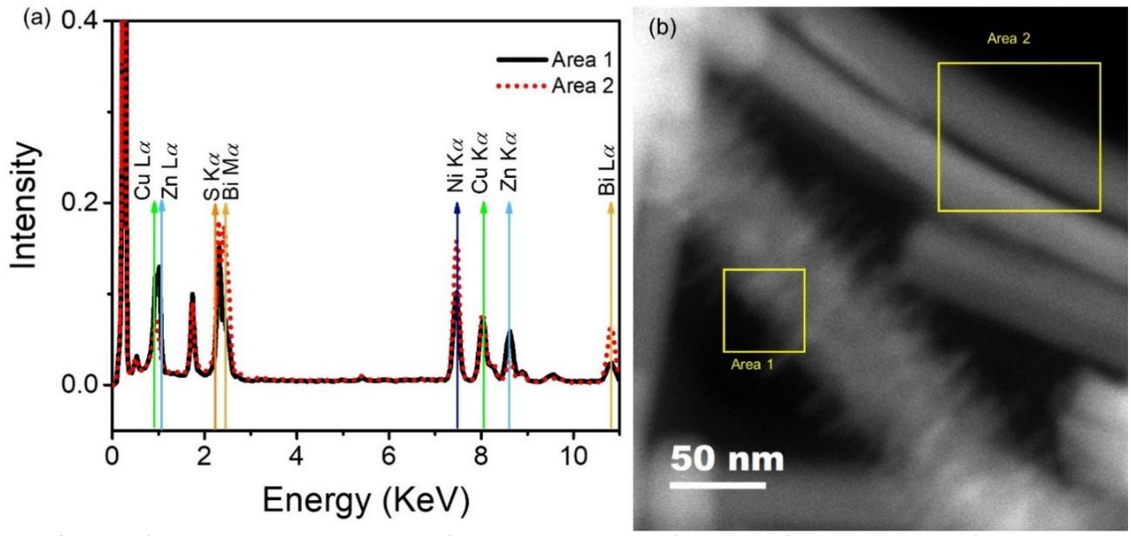

Figure S15. (a) Comparison elemental counts from selected area EDS spectra of the Zn rich area of the NRs with ZnS protrusion and the alloyed NRs shown in (b) STEM-ADF micrograph of the NRs alongside the NRs with ZnS protrusions.

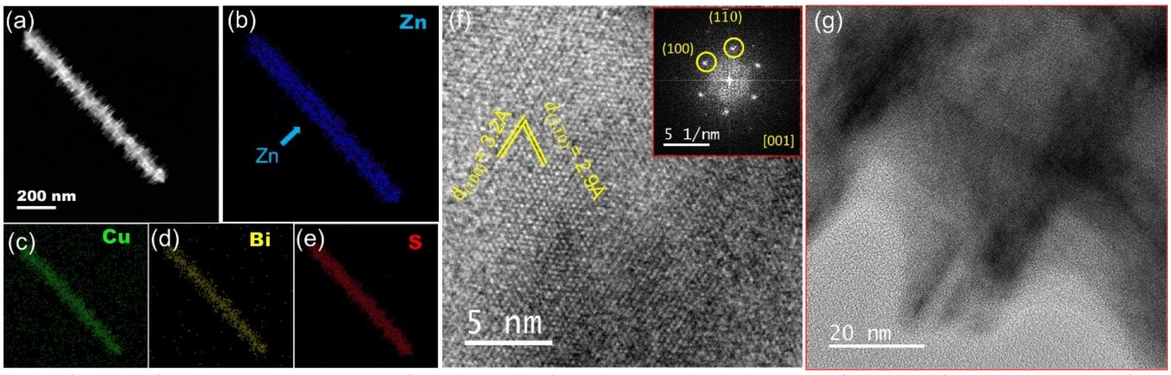

Figure S16. (a) STEM-ADF micrograph of a NR with ZnS protrusions accompanying STEM-EDS elemental maps for (b) Zn(Ka), (c) Cu(Ka), (d) Bi (La), (e) S (Ka). (f) HRTEM of the ZnS protrusion with insets displaying the FFT; (g) TEM image of ZnS protrusion on the NRs.

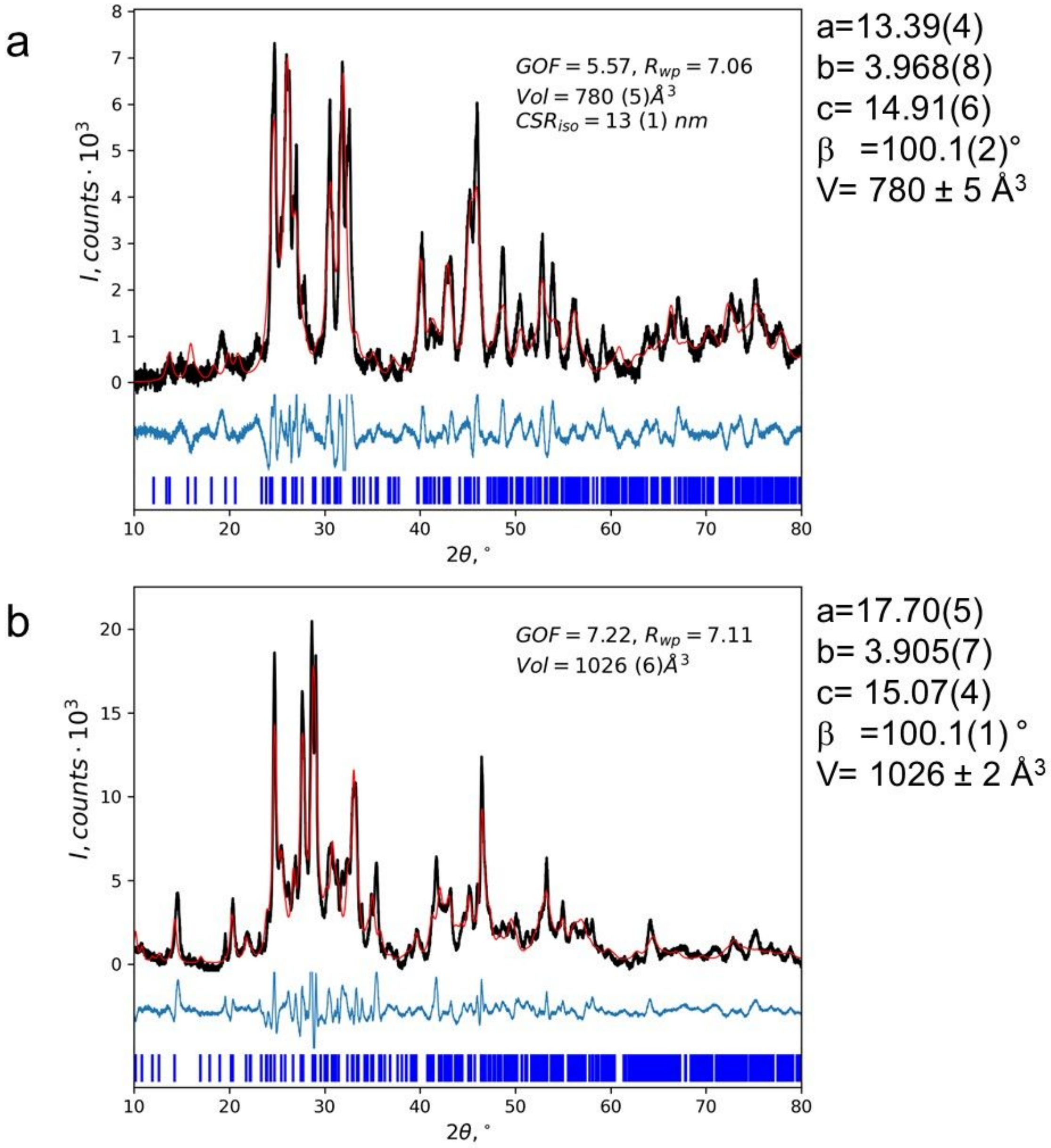

Figure S17. (a) Rietveld refinement and lattice parameters of the experimental XRD pattern of NR1 in black with red spectra depicting the calculated pattern and blue curve presents the difference spectra; (b) Rietveld refinement and

lattice parameters of the experimental XRD pattern of NR2 in black with red spectra depicting the calculated pattern and blue curve presents the difference spectra. For NR1 PDF No. 03-065-5469 and for NR2 PDF No. 03-065-4965 were used as reference.

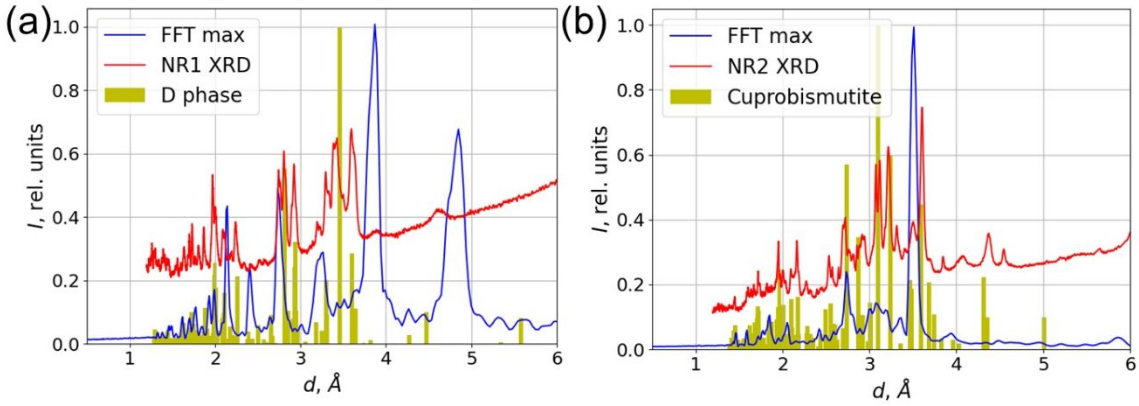

Figure S18. (a) Overlaid spectra of FFT intensity maximum, XRD pattern of NR1 and simulated XRD of $Cu_{2.94}Bi_{4.8}S_9$ (D-phase, PDF No. 03-065-5469); (b) (a) Overlaid spectra of FFT intensity maximum, XRD pattern of NR2 and simulated XRD of $Cu_{10.44}$ $Bi_{12.57}$ S_{24} (cuprobismutite, PDF No. 03-065-4965).

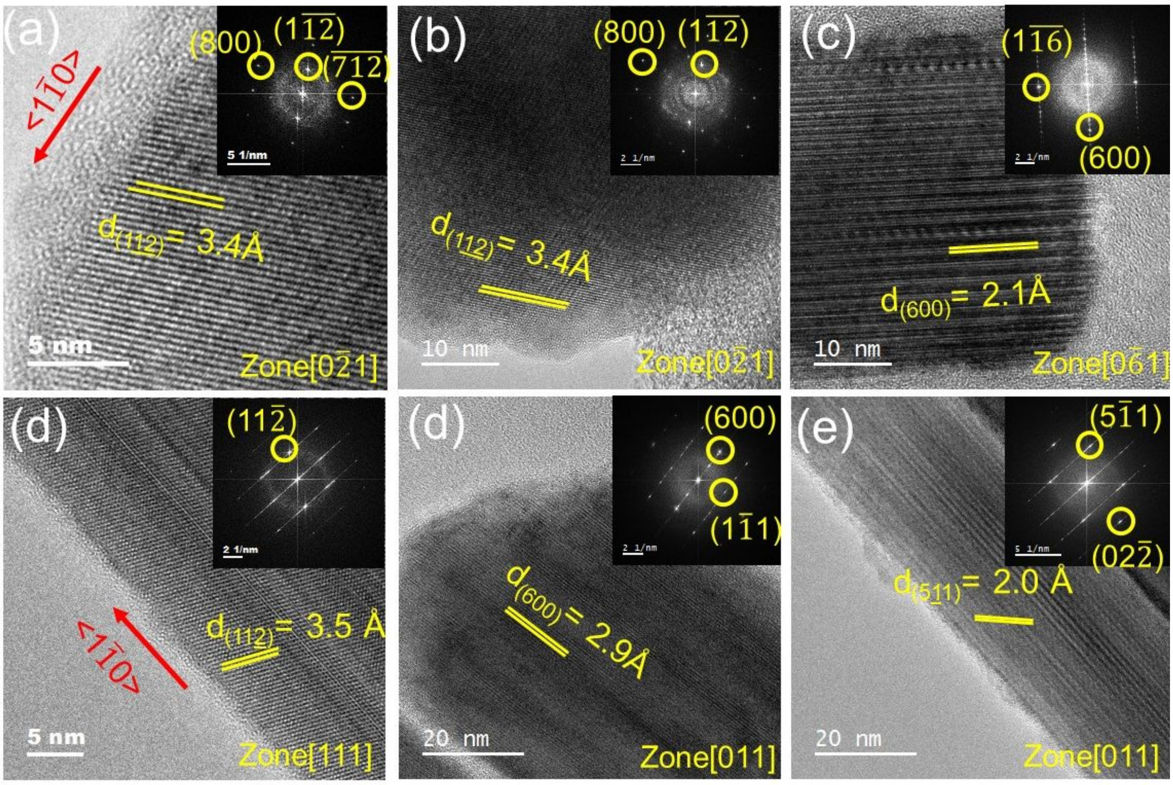

Figure S19. (a-c) HRTEM of NR1 sample accompanying FFT patterns in inset which match well with $Cu_{2.94}Bi_{4.8}S_9$-phase; (d-e) HRTEM of NR2 sample accompanying FFT patterns in inset which match well with cuprobismutite.

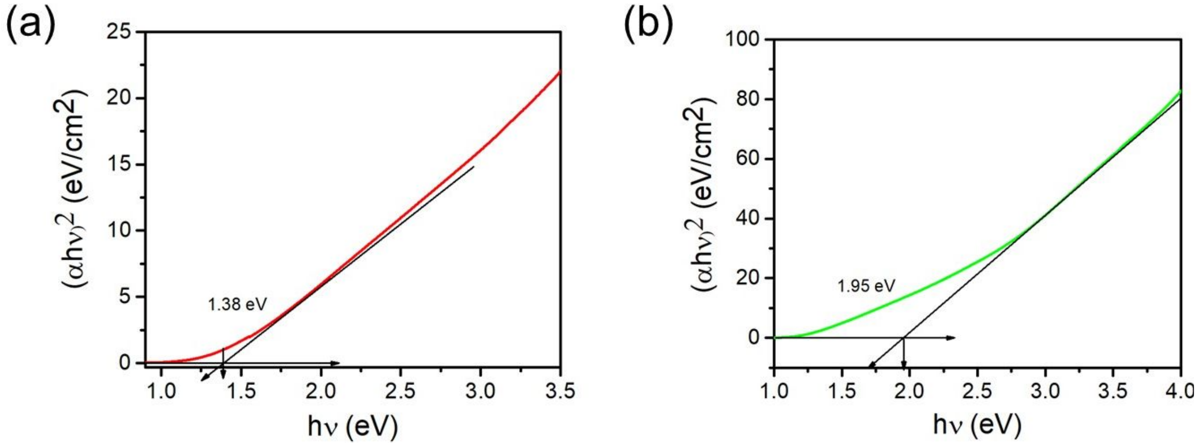

Figure S20. Tauc plot of (a)Zn poor NR1 and (b) Zn rich NR2**.**

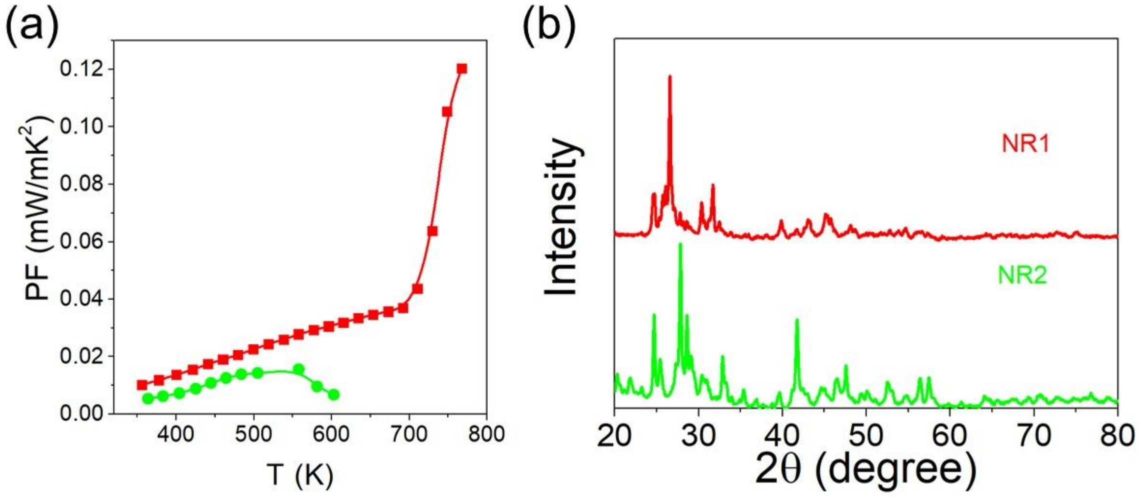

Figure S21. (a) Power factor of NR1 (red square) and NR2 (green circle); (b) XRD pattern of the NR pellets after TE measurements.

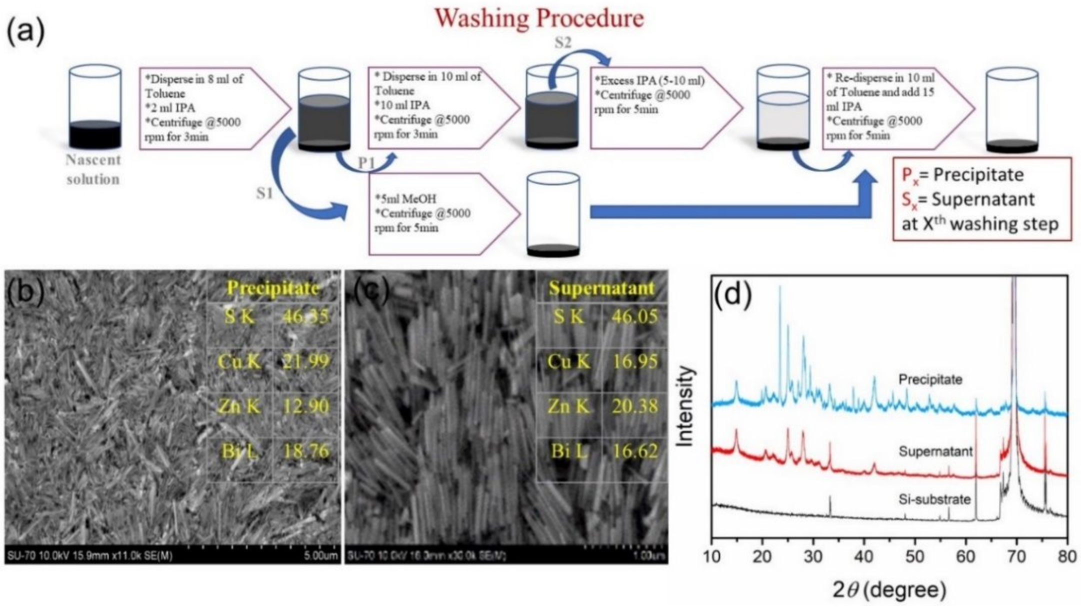

Figure S22. (a) Schematic description of the washing procedure for NCs purification; (b) SEM of the NCs present in the precipitate; (c) SEM of the purified NCs present in supernatant; (b) Comparison of XRD pattern collected from precipitate and supernatant NCs drop casted onto Si-substrate.

Effect of TOPO:
Trioctylphosphine oxide (TOPO) was used because of its ability to increase the solubility of metal precursors as a co-ordinating solvent in the colloidal systemFurthermore, in our case, changing the TOPO concentration has no effect on the morphology of the nanorods as shown in Figure S21. However, the nanorods synthesized without TOPO display poor colloidal stability with wide aggregation compared to the optimized conditions with the use of TOPO.

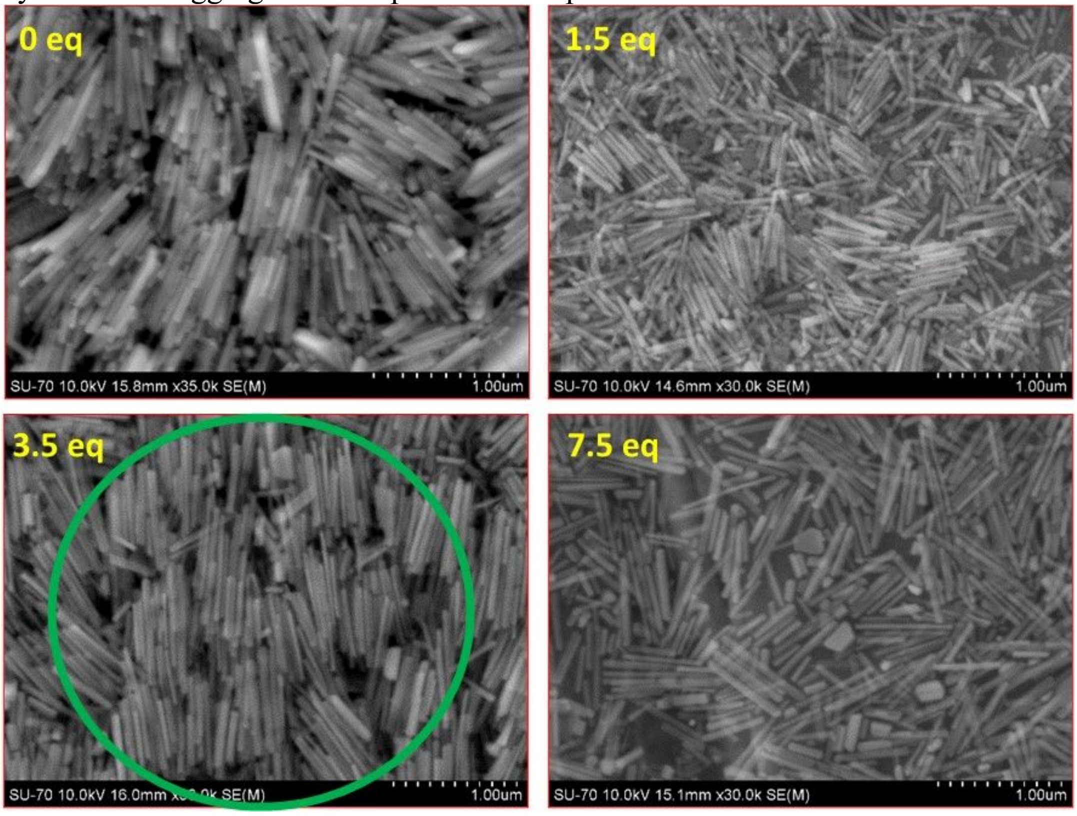

Figure S23. Scanning electron micrographs of Cu-Bi-Zn-S NRs using different concentrations of Trioctylphosphine oxide (TOPO). Highlighted concentration has been used in the main synthesis.

Effect of thiol mixture: Alkanethiols are soft Lewis base with a strong affinity for Cu^+ cations in colloidal reaction media. Compared to bulky *tert*-dodecanethiol (t-DDT), linear 1-dodecanethiol (1-DDT) shows stronger affinity for Cu^+ which slows the nucleation kinetics. Hence, mixing sterically hindered t-DDT helps to regulate the reactivity of Cu^+ during nucleation and growth. It should be noted the decomposition temperature of 1-DDT is ~ 280 °C compared to ~230 °C for t-DDT. Hence, a thiol mixture containing more t-DDT will release more active S for nucleation. Additionally, by modulating the concentration of 1-DDT in the thiol mixture, we observed significant changes in the shape of the NCs. By using only *t*-DDT, the NRs are formed with few NRs displaying higher width (Figure R2a, b). While increasing the amount of 1-DDT to more than 50 % in the thiol mixture, the morphology of the NCs changes to nail shape (50% 1-DDT, Figure R2c) and multipods (100 %, 1-DDT Figure R2d). Hence, it can be said insufficient passivation of facets by t-DDT resulted in wider NRs when zero concentration of 1-DDT is used. In comparison, the higher decomposition temperature of 1-DDT, higher Cu binding affinity, and high passivation resulted in irregular shape of the NCs when a high amount of 1-DDT is used.

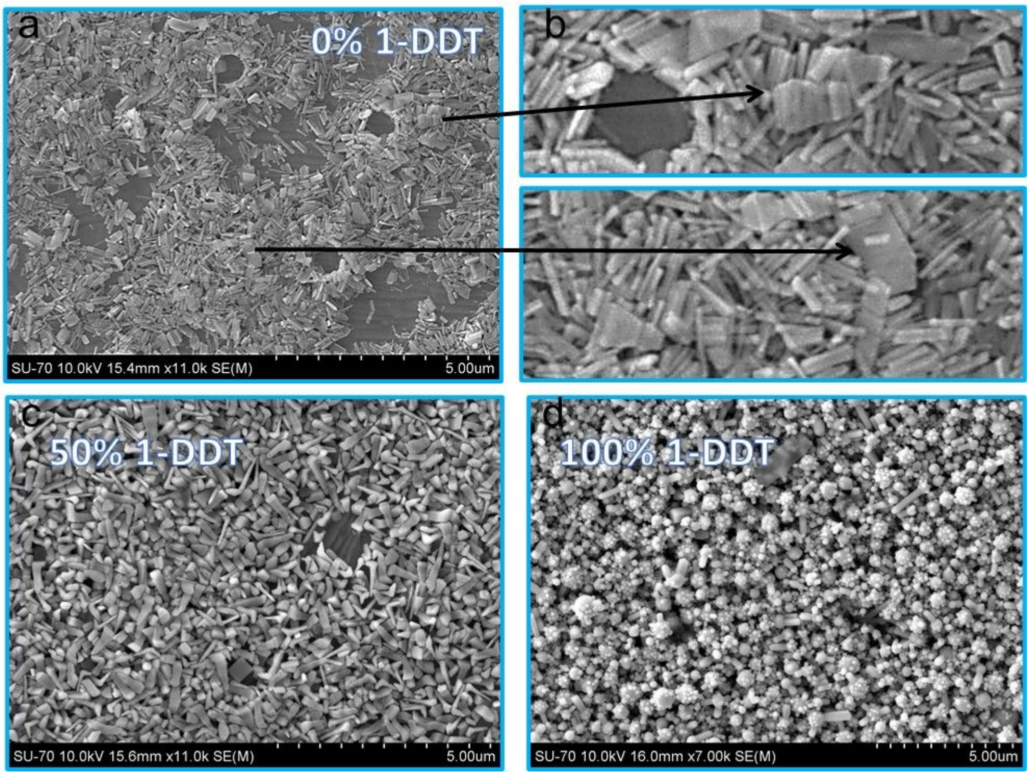

Figure S24. Scanning electron micrographs of Cu-Bi-Zn-S NCs using different concentration of 1-DDT (a) 0%, (b) highlighted wide NRs, (c) 50% and (d) 100%, in t-DDT and 1-DDT mixture keeping the total amount 1 ml as used in optimized synthesis.

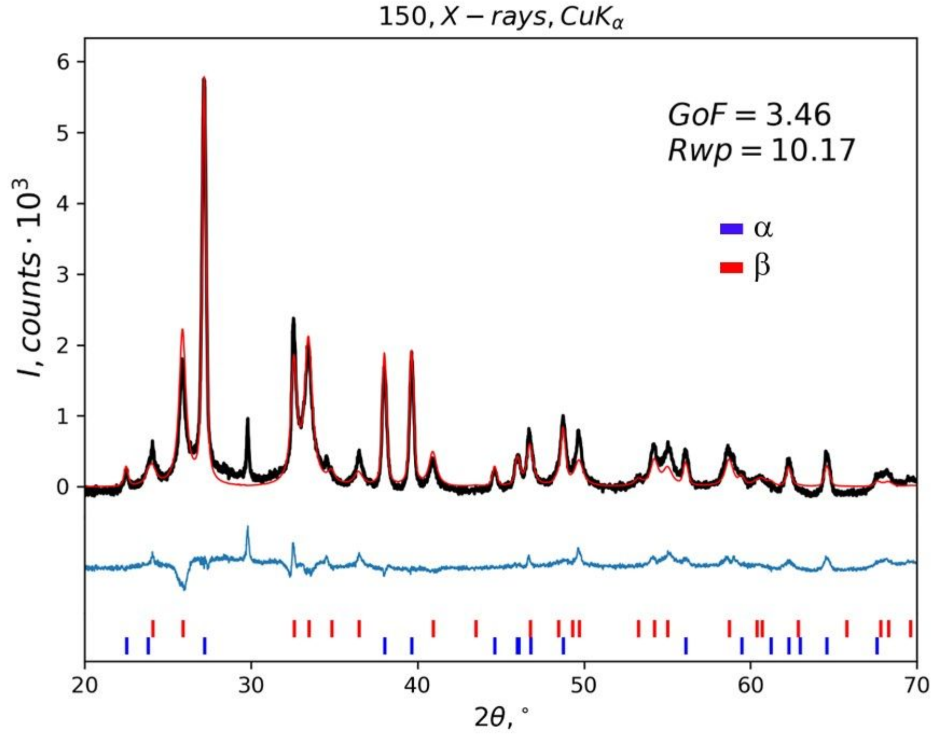

Figure S25. Rietveld refinement of the experimental XRD pattern of the NCs collected from 150 °C aliquot in black with red spectra depicting the calculated pattern and blue

curve presents the difference spectra. For Bi, denoted as α (PDF No. 04-003-1496), and BiOCl, denoted as β (PDF No.00-006-0249) reference were used.

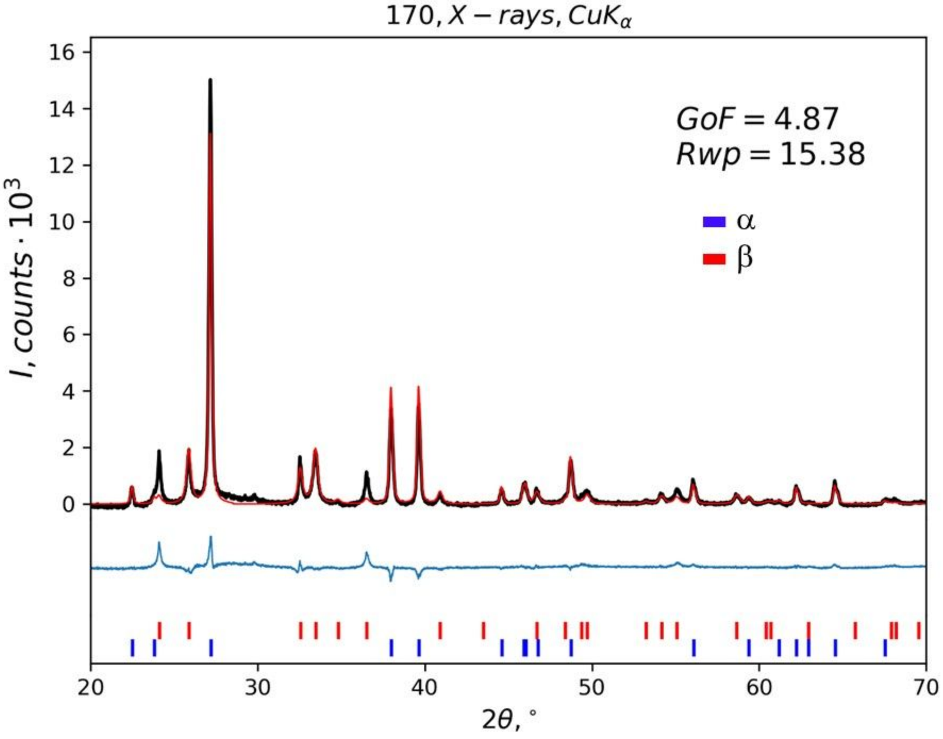

Figure S26. Rietveld refinement and lattice parameters of the experimental XRD pattern of of the NCs collected from 170 °C aliquot in black with red spectra depicting the calculated pattern and blue curve presents the difference spectra. For Bi, denoted as α (PDF No. 04-003-1496), and for BiOCl, denoted as β (PDF No.00-006-0249) reference were used.

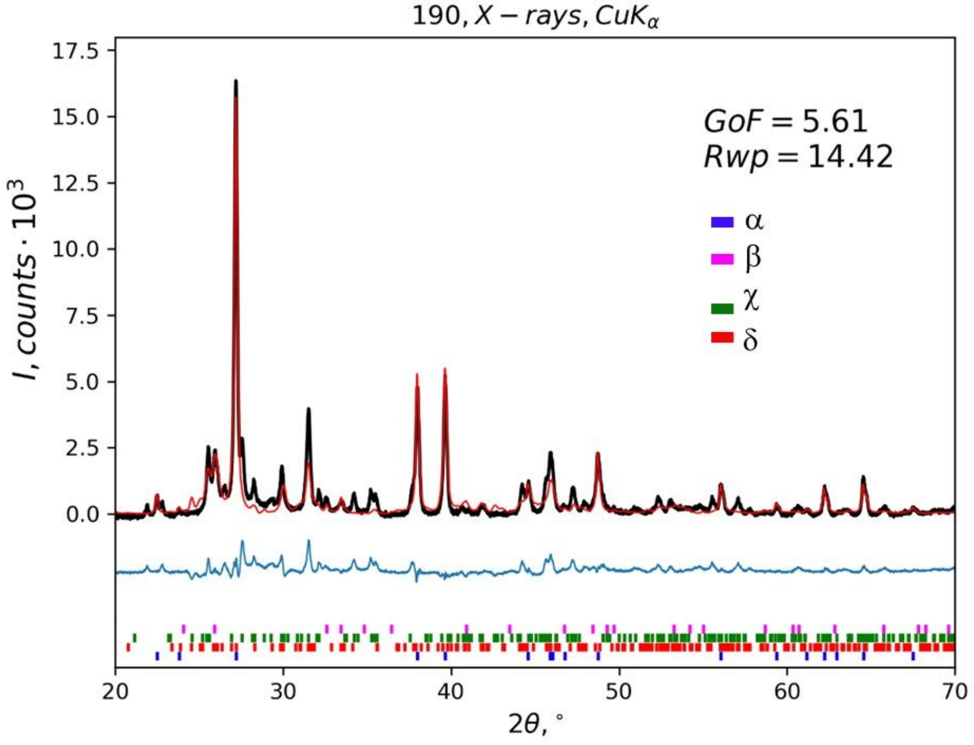

of the NCs collected from 190 °C aliquot in black with red spectra depicting the calculated pattern and blue curve presents the difference spectra. For Bi, denoted as α (PDF No. 04-003-1496), for BiOCl, denoted as β (PDF No.00-006-0249) reference were used.

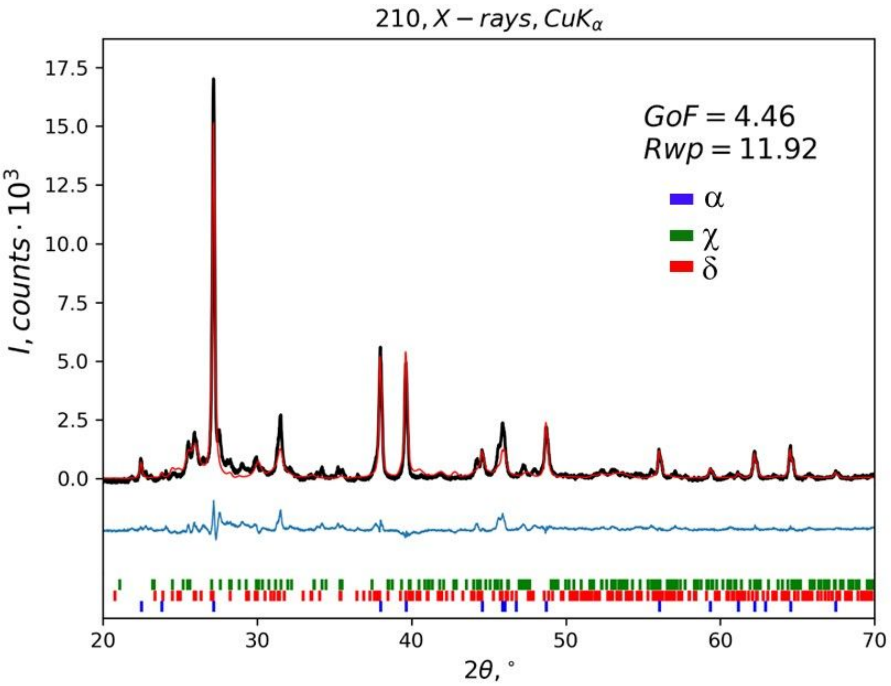

Figure S28. Rietveld refinement and lattice parameters of the experimental XRD pattern of of the NCs collected from 210 °C aliquot in black with red spectra depicting the calculated pattern and blue curve presents the difference spectra.

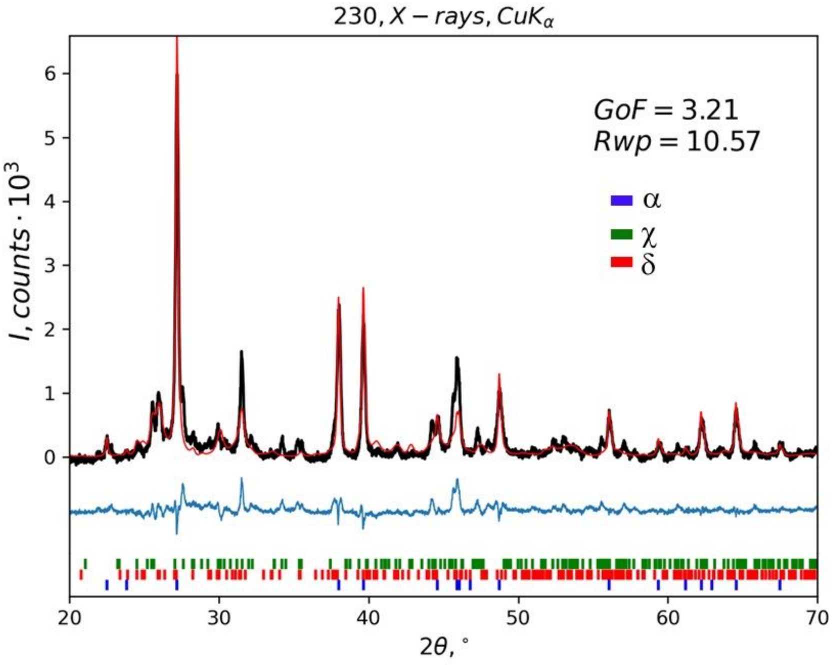

Figure S29. Rietveld refinement and lattice parameters of the experimental XRD pattern of of the NCs collected from 230 °C aliquot in black with red spectra depicting the calculated pattern and blue curve presents the difference spectra.

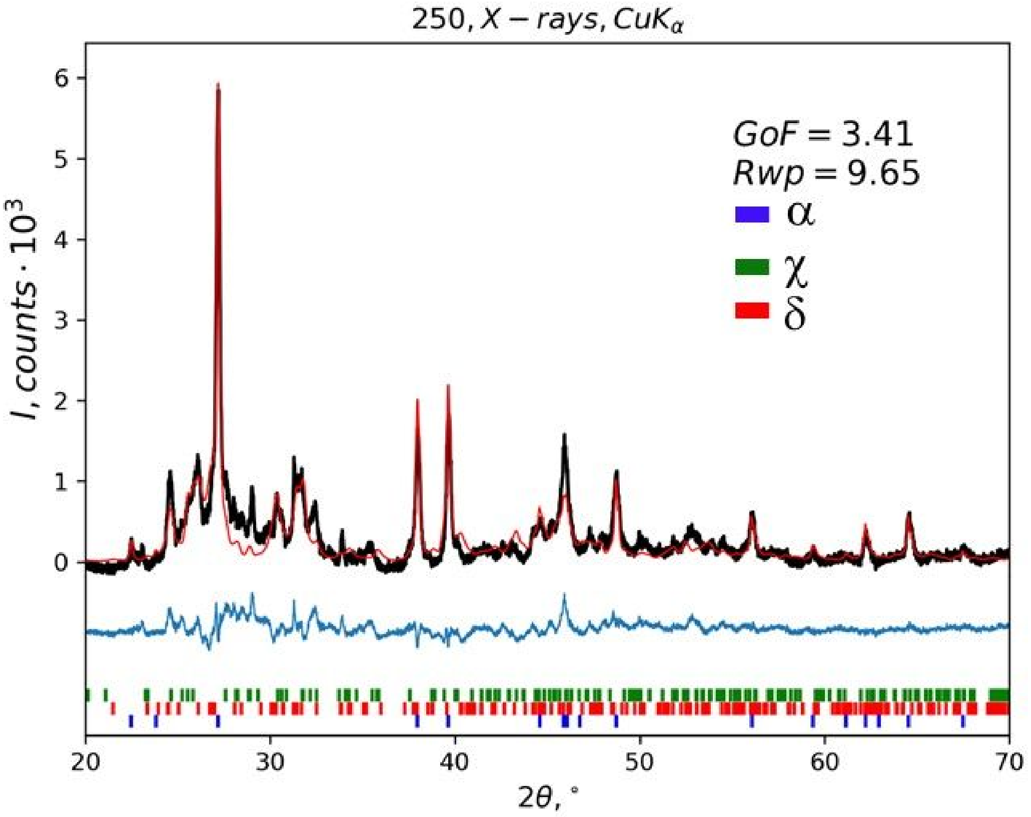

Figure S30. Rietveld refinement and lattice parameters of the experimental XRD pattern of of the NCs collected from 250 °C aliquot in black with red spectra depicting the calculated pattern and blue curve presents the difference spectra.

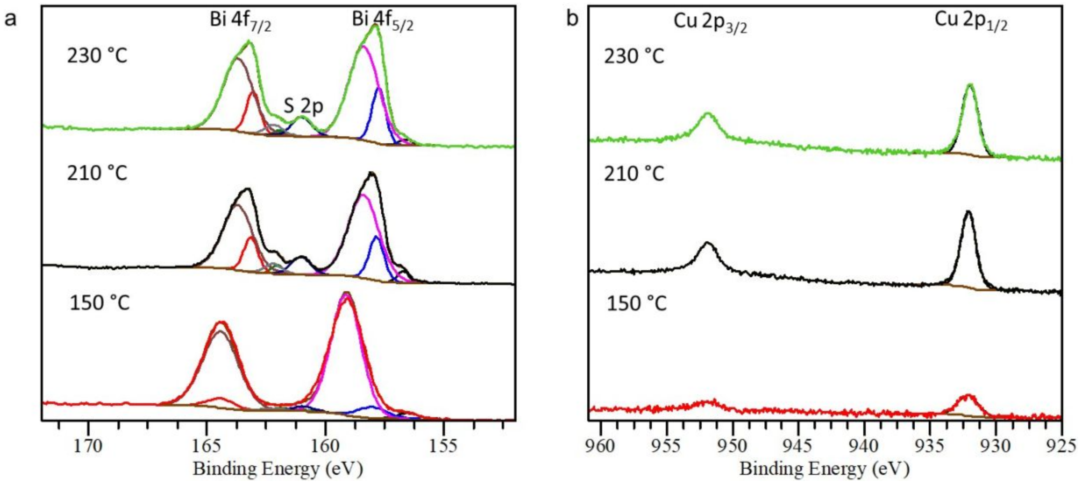

Figure S31. XPS analysis of the solid products derived from the aliquots collected at 150, 210, 230 °C. (a) Bi4f and S2p spectra, the peaks at ~ 156.5 and 161.8 eV correspond to metallic bismuth of the seed and the peaks at ~161 and ~162 eV corresponds to sulphide from metal sulphide bond ($Cu_{2-x}S$ and $Bi_xCu_yS_z$) for all temperatures. The peaks near 163.8 and 158.9 eV belong to Bi^{3+} of Bi-S bond for 210, and 230 °C aliquot, which is absent in 150 °C suggesting the absence of any $Bi_xCu_yS_z$ phase. For the 150 °C aliquot, the peaks of 164.5 and 159.2 eV belong to Bi^{3+} from $BiCl_3$ or BiOCl, which might have formed from the exposure of unreacted $BiCl_3$ to air and moisture.

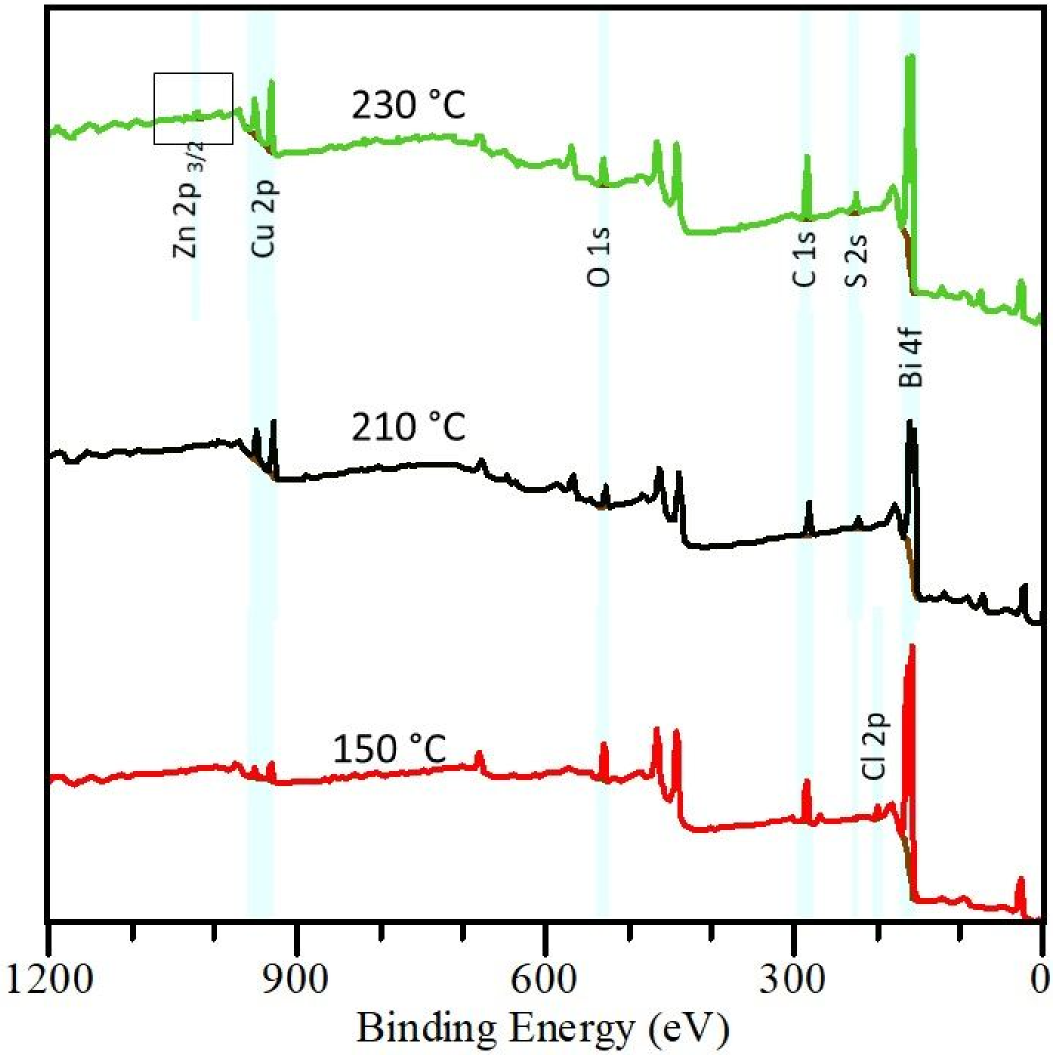

Figure S32. XPS survey of the solid products derived from the aliquots collected at 150, 210, and 230 °C. The XPS survey exhibited a peak for Zn 2p around 1045 eV suggesting the presence of Zn^{2+} in NCs collected at 230 °C; however, no Zn^{2+} signal was found from the aliquot collected at 210 °C further corroborating our observation that Zn^{2+} will come into effect only after 230 °C.

Liquid phase analysis:

The supernatant of the aliquot collected at 150, 210, and 230 ˚C were characterised using FTIR. The -C=C-H (~3050 cm^{-1}), C-H alkyl stretching (~2920 cm^{-1}symmetric and 2850 cm^{-1} asymmetric) belongs to oleylamine and 1-ocatdecene present in the liquid. It was further supported by the presence of -C-N stretching mode near ~1120 cm^{-1} and C=C stretching mode at 1645 cm^{-1}. The C-S stretching mode is present near ~700 cm^{-1}. However, S-H stretching mode is absent near ~2500 cm^{-1} suggesting the removal of S-H due to the interaction of alkane thiols with cationic sources and thermolysis of alkanethiol to produce active sulfide species. Similarly, the stretching mode of P=O displayed a broad peak ~1150 $cm^{-1,}$ suggesting the presence of TOPO.

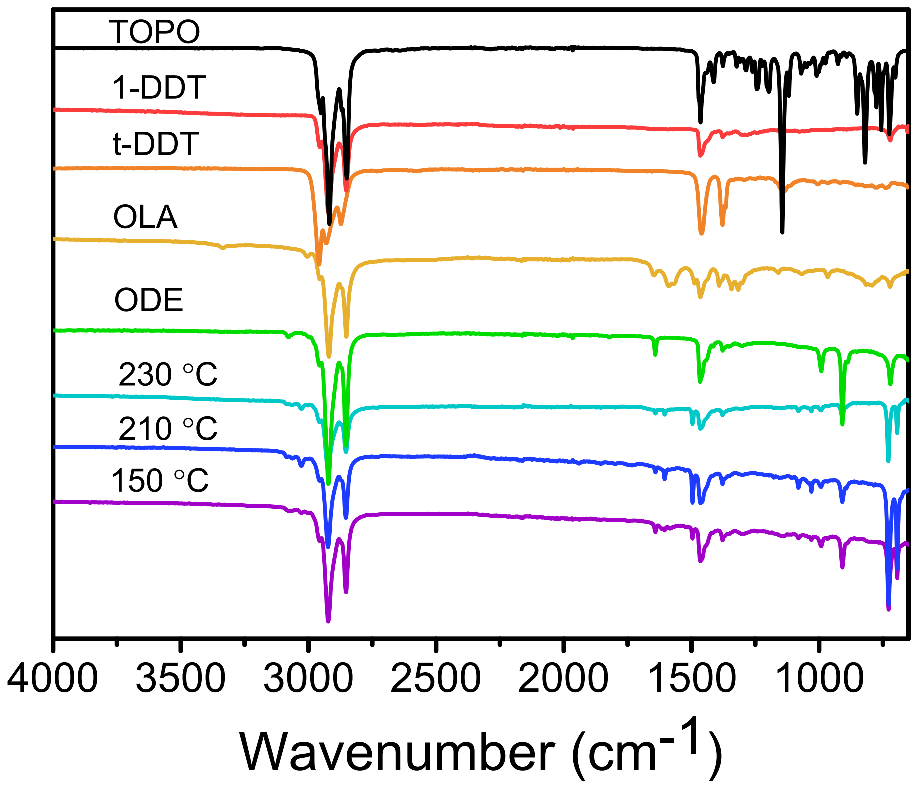

Figure S33. FTIR analysis of aliquot supernatants.
